# What happens when we modify mosquitoes for disease prevention? A systematic review

**DOI:** 10.1080/22221751.2020.1722035

**Published:** 2020-02-11

**Authors:** Teresa Nazareth, Isabel Craveiro, Alanny Moutinho, Gonçalo Seixas, Cátia Gonçalves, Luzia Gonçalves, Rosa Teodósio, Carla A. Sousa

**Affiliations:** a UEI Parasitologia Médica, Global Health and Tropical Medicine (GHTM), Instituto de Higiene e Medicina Tropical (IHMT), Universidade Nova de Lisboa (UNL), Lisboa, Portugal; b UEI Bioestatística e Sáude Internacional, Global Health and Tropical Medicine (GHTM), Instituto de Higiene e Medicina Tropical (IHMT), Universidade Nova de Lisboa (UNL), Lisboa, Portugal; c Nova School of Business and Economics, Universidade NOVA de Lisboa, Lisboa, Portugal; d Centro de Estatística e Aplicações, Universidade de Lisboa, Lisboa, Portugal; e UEI Clínica Tropical, Global Health and Tropical Medicine, (GHTM), Instituto de Higiene e Medicina Tropical (IHMT), Universidade Nova de Lisboa (UNL), Lisboa, Portugal

**Keywords:** Vector-borne diseases, transgenesis, Wolbachia, genetically modified mosquitoes

## Abstract

The release of modified mosquitoes to suppress/replace vectors constitutes a promising tool for vector control and disease prevention. Evidence regarding these innovative modification techniques is scarce and disperse. This work conducted a systematic review, gathering and analysing research articles from PubMed and Biblioteca Virtual em Saúde databases whose results report efficacy and non-target effects of using modified insects for disease prevention, until 2016. More than 1500 publications were screened and 349 were analysed. Only 12/3.4% articles reported field-based evidence and 41/11.7% covered modification strategies’ post-release efficacy. Variability in the effective results (90/25.7%) questioned its reproducibility in different settings. We also found publications reporting reversal outcomes 38/10.9%, (e.g. post-release increase of vector population). Ecological effects were also reported, such as horizontal transfer events (54/15.5%), and worsening pathogenesis induced by natural *wolbachia* (10/2.9%). Present work revealed promising outcomes of modifying strategies. However, it also revealed a need for field-based evidence mainly regarding epidemiologic and long-term impact. It pointed out some eventual irreversible and important effects that must not be ignored when considering open-field releases, and that may constitute constraints to generate the missing field evidence. Present work constitutes a baseline of knowledge, offering also a methodological approach that may facilitate future updates.

## GRAPHICAL ABSTRACT

**Figure:**
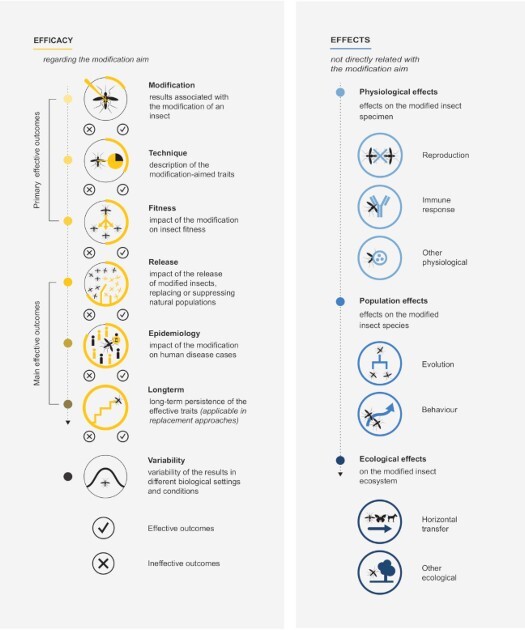

## Introduction

Vector-borne diseases have a wide impact on human health being a mandatory topic on global health agendas [1,2]. Even with the significant reduction of the global burden of malaria since the beginning of the century, in 2016, this infectious disease was still responsible for 445,000 deaths [3]. Due to human population growth, globalization, and climate change, arboviral diseases outbreaks have been increasing in frequency, expansion, diversity, and severity [4]. Only dengue’s incidence grew more than 30-fold in the last 50 years [5]. Although arboviruses dispersal is partially conditioned by the environmental constraints that limit the distribution of its main vectors, outbreaks of diseases such as yellow fever, chikungunya, and Zika have been reported all over the world [6–10]. The severity of Zika fetal malformations during 2015/2016 epidemics turn it a public health emergency of international concern according to World Health Organization [8]. The lack of effective approved vaccines for some of these infections and the increase of insecticide resistance in its most competent vectors, impose an urgent need for innovative effective strategies to minimize these diseases [9,10].

The release of modified insects is considered a promising approach for prevention and control of vector-borne diseases. Innumerous techniques and insects’ modification strategies had been laboratorial tested, all of them fitting one of the two broad approaches: (i) modification and release of sterile insects aiming the reduction/eradication of natural vector populations (suppression approach/vector control approach) or (ii) modification and release of insects refractory to pathogen transmission aiming the replacement of natural vector populations (replacement approach/transmission prevention approach). Open releases of modified insects have been occurring all over the world in an attempt to cope the unprecedented vector-borne diseases burden [11]. However, none of these modifying technologies has yet been approved by the WHO’s Vector Control Advisory Group [12].

Few studies reported the effectiveness of mosquito modification strategies, and even less their eventual effects exploring them only barely and theoretically [13]. Important reviews on this topic were recently published, but corresponding to the perspective of the author regarding the subject or a summary of the authors’ selection of publications [14,15]. This work presents a unique structured review on the use of modified insects to control and prevent vector-borne diseases, gathering, exploring, and classifying evidence available up to 2016 regarding efficacy and (non-target) effects of these modifying techniques.

## Methods

The present work is enrolled in a bigger project whose aim is the description of the strengths/weaknesses/opportunities/threats of modified insects for disease prevention (genetic, radiation-based, or other modifications). During analysis and reviewers’ consensus, it was realized that all evidence found constituting strengths/weaknesses/opportunities/threats of the insects’ modification for disease prevention were fitting in two main themes: the efficacy and the non-target effects of the insects' modifications. Hence, results were extracted and are presented according to this classification.

### Search strategy

To identify relevant documents focusing on strengths, weaknesses, opportunities, and threats of modifying insects to prevent diseases, two electronic databases (PubMed and Biblioteca Virtual em Saúde, BVS) were searched using combinations of MeSH terms and free text words such as “organisms, genetically modified” (MeSH), “wolbachia,” “lethal,” “sterile insect,” “vector-borne,, “replacement,” and “suppression.” To help increase sensitivity and specificity, combinations of different search strings were used for each electronic database. Results from all searches were downloaded into Mendeley program (Elsevier); duplicates were withdrawn automatically using Mendeley and verified manually, followed by the inclusion process implementation.

### Study selection

Publications were included in the study when all of the following inclusion criteria were met:

1. Research articles, i.e. publications structured as Introduction, Material and Methods, and Results/Discussion, or similar.
2. Available as Free Full-Text at NOVA Discovery platform.
3. Written in English, French, Portuguese, or Spanish.
4. Published until the date of the search (1 March 2016).
5. Publications covering modified insects or the modifications itself. It was considered “modification” any process, species, or condition, described in the literature as able to be used to modify insects (rather genetic or other type of modification), even if not explicit in the collected paper.
6. Publications whose results explicitly report strengths, weaknesses, opportunities, and threats of the modifications (or eventual modifications) concerned with health impact and/or biological impact
7. Publications whose studies were performed in Insect or Mammal species, rather *in vitro*, *in vivo*, *ex vivo*, or in archetypal modelled species.
  These inclusion criteria (point 5) cover publications regarding several types of modifications, such as *Wolbachia*-based, radiation-based, genetic-based, etc. and in the case of ‘*Wolbachia’* search term it included both natural or artificial *Wolbachia* infections.
  Also, even tough most work on this area has been done for mosquito-borne diseases’ prevention, the term ‘insects’ were selected instead of ‘mosquitoes’ (point 7). This criteria aims to not exclude publications reporting modifications often applied to mosquitoes when applied to other insects, namely for laboratory ease and also to include non-mosquitoes insect vectors such as Glossinas spp.

A two-stage inclusion process was applied. All references were initially screened by title and abstract and included in the study if they met the selection criteria. In the second stage, the full-text reading of each publication was undertaken. To establish consensus in criteria application, part of the publications (5% of the 1st screening, and 50% of the 2nd screening) were screened by two reviewers (inter-reviewer check). Disagreements were resolved by discussion. At the end of the 2nd screening and after criteria had been discussed, the full-text screening was repeated by one reviewer to ensure homogeneity of the criteria during the process (temporal check). All documents considered relevant went to the next phase of extracting data and analysis.

### Data synthesis and analysis

Data were extracted from the included publications into a digital data-extraction form. Two investigators performed data extraction and analysis of 50% of the included publications (inter-reviewer check). All extracted data were structured into two major themes, efficacy and effects of the modification strategies, and each of them divided into several topics and sub-topics (see more detailed information in Results section). These hierarchical categories were defined by the two reviewers through a consensual process. Disagreements were resolved by discussion. When consensus was attained, categorization and analysis of all included articles were re-checked in order to ensure a homogeneous analysis (temporal check). According to the evidence reported, publications were classified into as many categories as possible, in order to reduce the likelihood of missing key points in the data. It was also extracted information regarding the year, type of study (laboratory, semi-field, field, and computational modelling), species involved in the experiments, and modification strategy (*Wolbachia*, anti-pathogen Transgenesis, lethal Transgenesis, etc.). As to *Wolbachia*-based studies, publications were classified according to the endosymbiont origin: natural occurrence, artificially introduced or removed from natural or artificially infected hosts. The classification regarding the type of study was used as a *proxy* of the publication robustness, considering semi-field and field studies the most robust ones, and computational modelling and laboratorial the less robust. Apart from qualitative analysis, descriptive statistics analysis was performed. The softwares Excel (Microsoft Office, Windows 10) and NVivo 10 (QSR International Pty Ltd, Doncaster, Victoria, Australia) were used during the analysis. Results are presented by theme, modification strategy and species, publications are also referred according to their chronological order on the manuscript’s sections, tables, and supplementary information. This literature review followed the proceeding of a PRISMA methodology (S1 Checklist).

## Results

**Figure 1.**
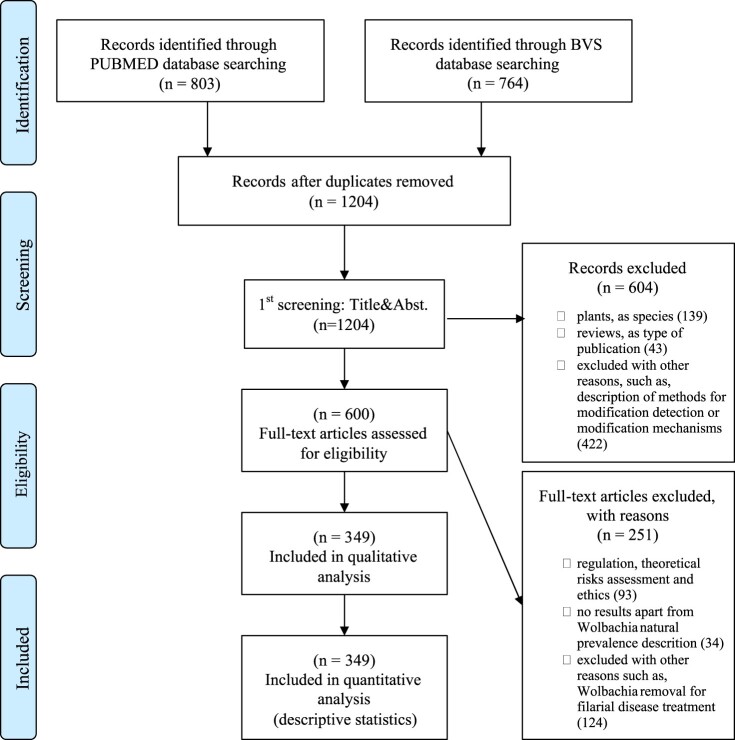
PRISMA Flowchart reporting the number of publications in each stage of the review.

Databases searches resulted in a total of 1567 publications (Figure 1). Following the removal of duplicates, 1205 references were selected. After the two-stage selection process, 377 articles were included in the study and 349 publications were analysed. References from analysed publications were ordered from 1 up to 349 and cited in *italic* for differentiation from manuscript’s references listed in the end of the manuscript (see full list of analysed publications and summary of analysis in S1 Appendix).

From the 349 publications selected for analysis, 340/97.1% were published after the year 2000. The majority constituted laboratory studies, i.e. performed in a controlled experimental environment (310/88.6%) and referred to *Wolbachia* or other symbiont-based modification strategy (307/88.0%). Several organisms’ species integrated in the experiments of the analysed publications: five genera of insects vectors (*Aedes, Anopheles, Culex, Mansonia, Phlebotomus*, and *Glossina*), 54 genera of non-vector insects, and four mammals genera (see all data regarding quantitative analysis in S1 Figure).

**Figure 2.**
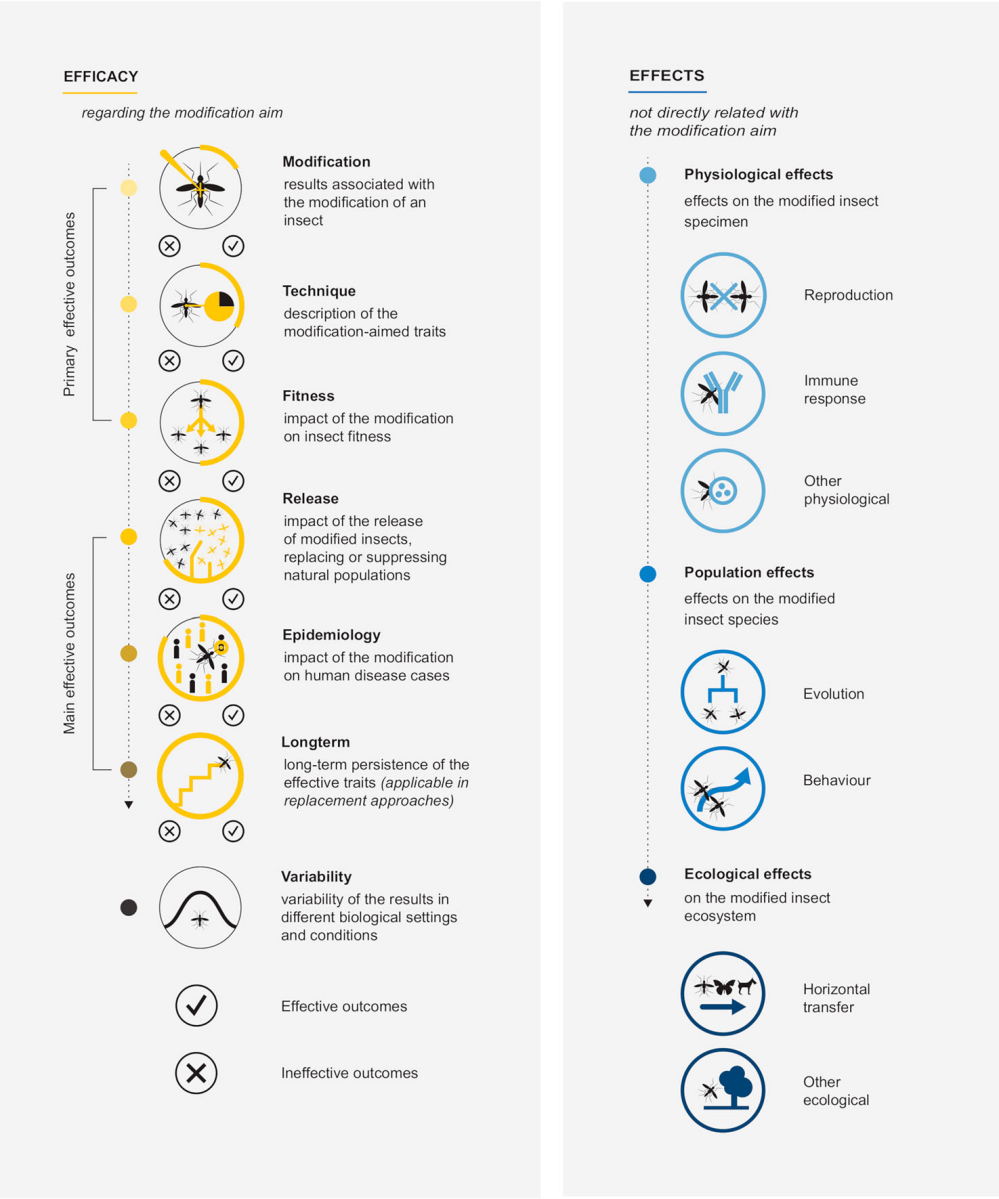
Schematic representation of the themes, topics, and type of outcomes described in the “Results” section.

In what concerns the content of the publications, two major themes emerged from the analysed research articles: (i) efficacy of the modification strategies and (ii) non-target effects induced by the modifications. Both themes (efficacy and effects) were divided into several topics (see Figure 2).

**Figure 3.**
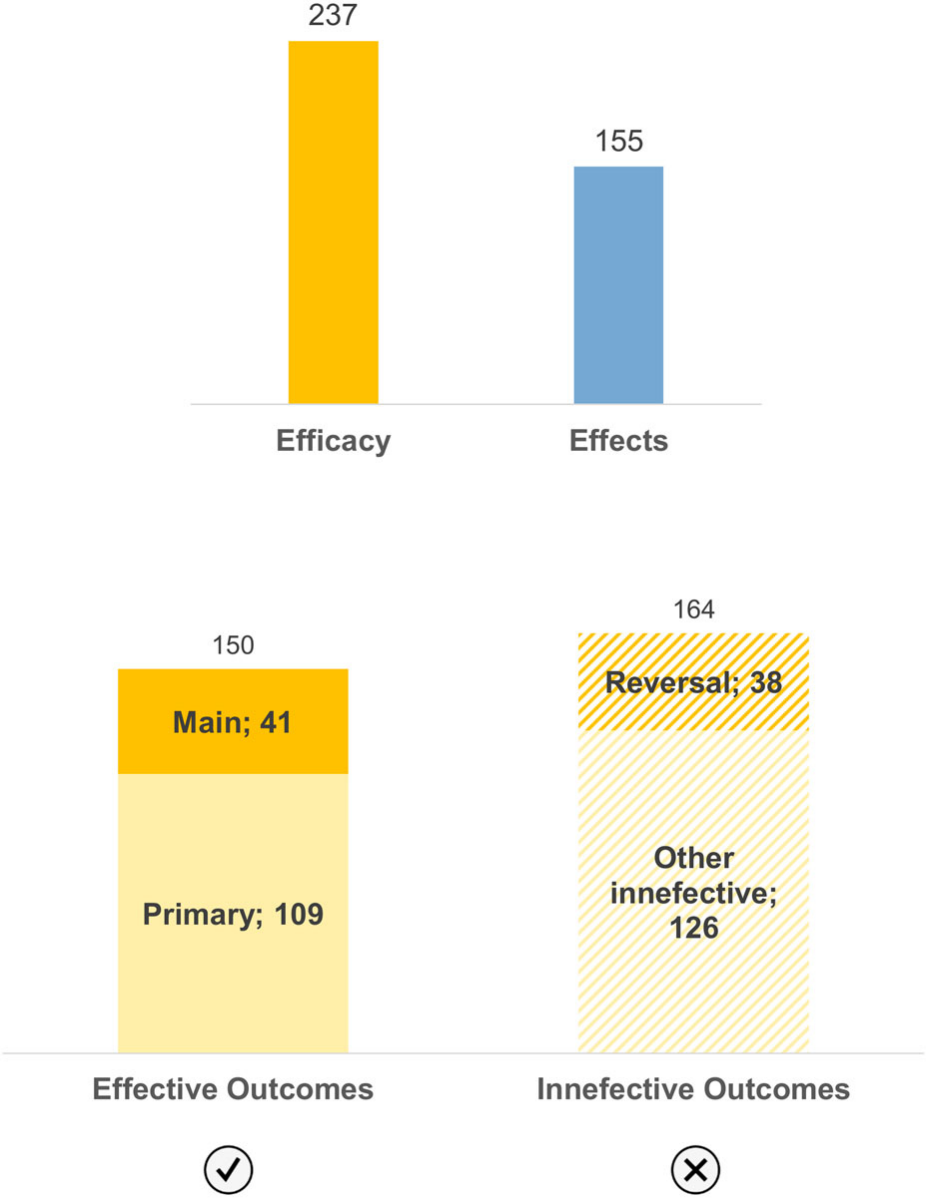
Distribution of the: (i) (above) publications whose results contributed to each of the major themes (*n *= 349); (ii) (below) publications whose results contributed to each type of outcomes in efficacy (According to Figure 2 Primary covering Modification, Technique, and Fitness topics, and Main covering Release, Epidemiology, and Long-term topics) (*n* = 237).

Efficacy outcomes were also divided into effective outcomes – reporting the success of the modification strategy – and ineffective outcomes – reporting the failure of the modification strategy. Ineffective outcomes include outcomes that (i) achieved no results, (ii) described indirect results that call into question the efficacy of the modification strategy, and/or (iii) reported reversal results, i.e. that lead to the reverse of the aim of the modification strategy. There were more publications contributing to efficacy (237/67.7%) than publications with results regarding effects (156/44.6%) (Figure 3). Regarding themes and topics’ analysis, each publication may have contributed to more than one category.

In what concerns publications covering efficacy outcomes, there were more publications reporting ineffective outcomes (164/69.2%) than publications reporting effective ones (150/63.3%). Out of the latter, only 41/27.3% constitute main effective outcomes (regarding its release, epidemiologic and long-term efficacy), and out of the former, 38/23.2% constitute reversal outcomes (Figure 3).

Next sections summarize the analysis by themes and topics, and by modification strategies as follows: (i) *Wolbachia* and other symbiont-based modification strategies and (ii) transgenesis and other non-symbiont-based modification strategies. Some modifications are the combination of the two above types of modification strategies. Examples of those are (i) Paratrangenesis, i.e. the introduction of a transgene in a symbiont bacteria infecting the insect (rather than introducing the transgene in the genome of the insect itself) *(2)*; (ii) the simultaneous introduction of a *Wolbachia* and a transgene in the same organism *(3)* (both included in “*Wolbachi*a and other symbiont-based” section); and (iii) transgenesis using *Wolbachia* as gene drive *(4–9)* (included in “Transgenesis and other” section).

## Efficacy

### *Wolbachia* and other symbiont-based modification strategies (effective and ineffective outcomes)

**Figure 4.**
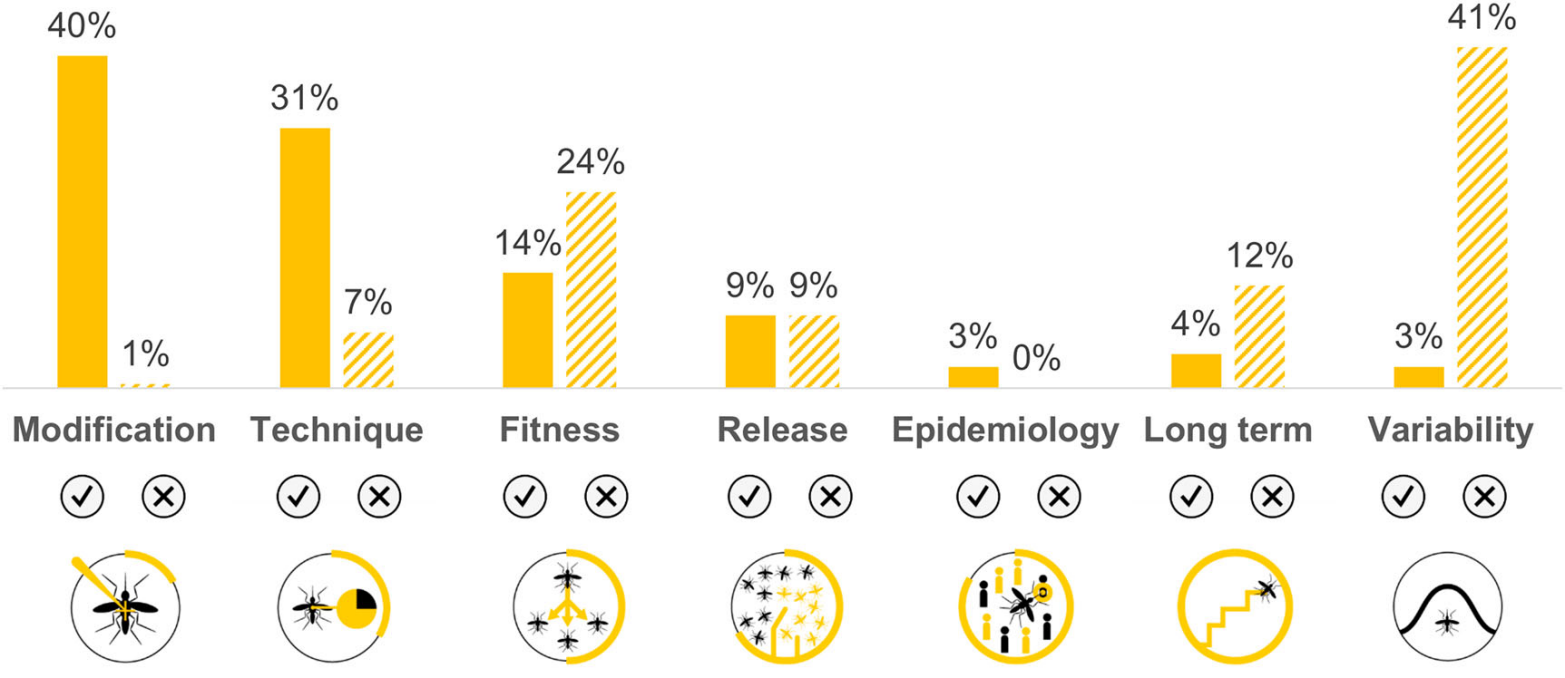
Distribution of the publications regarding Wolbachia and other symbiont-based modification strategies, whose results contribute to each efficacy topic, distinguishing effective/ineffective outcomes (*n* = 195).

A total of 195/82.3% research articles, out of the 237 with efficacy outcomes, presented results regarding the efficacy of *Wolbachia* or other symbiont-based insect modifications (111/56.9% reported effective outcomes, 145/74.4% reported ineffective outcomes, nTotal = 195). One publication reported the efficacy of other symbiont-based insect modification strategy (a *Sodalis* modified by paratrangenesis) *(2)*, and two publications referred to computational studies using archetypal modelled endosymbionts *(10),(11)*. Efficacy results covered all the topics (Modification, Technique, Fitness, Release, Epidemiology, Long-term, and Variability) as presented in Figure 4 and described in the following paragraphs. *Wolbachia*-related efficacy results were based on artificial *Wolbachia* infections in all topics except on variability topic which results came both from natural and artificial *Wolbachia* infections.

#### Modification

According to analysed publications, different *Wolbachia* strains were microinjected into several insect species, leading to a successful germline infection of the insect (stable transinfection). Stable transinfections were reported in the following insect species: *Aedes aegypti (12–31*)*, Aedes albopictus (32–36*)*, Aedes polynesiensis (37),(38), Anopheles gambiae (39*)*, Anopheles stephensi (40),(39),(41),* and *Culex tarsalis (42)* (vector mosquitoes), and *Bemisia tabaci* (whitefly) *(43)*, *Ceratitis capitata* (medfly*) (44), Drosophila melanogaster* (fruit fly) *(45),(46), Drosophila simulans* (fruit fly) *(47–50),(45),(51),(46),(52), Ephestia kuehniella* (butterfly) *(53),(54), Laodelphax striatellus* (planthopper) (*55)* (non-vector insects). Apart from transinfections, analysed articles also described other types of *Wolbachia* successful introduction such as transient somatic infection (*56–58)*, infections in cell lines *(59–65)*, ex *vivo* organ culture *(66)*, outcrossing *(67),(68)*, and introgression *(69–71),(45),(72),(19),(67),(73–77)*. One article reported the loss of *wMe*l on an *Aedes albopictus* cell line 12 passages post-infection *(61)*.

#### Technique

In what concerns the traits that ensure the *Wolbachia* effectiveness, several publications reported high cytoplasmic incompatibility (CI), i.e. the survival of offspring from infected females (>90%) and/or perfect (100%) maternal transmission, i.e. the transmission of *Wolbachia* to the insect host’s offspring, on vectors species mainly in *Aedine* species *(12),(17),(19),(68),(27),(30),(74),(78–80),(75),(37),* but also in *Anopheles stephensi (40),(39), and Culex pipiens quinquefasciatus (81),(82). Wolbachia*-induced pathogen protection was first reported in *Drosophilinae* species, protecting from infection of several RNA virus *(83)*. Later, pathogen protection induced by artificial introduction of *Wolbachia* was also demonstrated in several vector species: not only in those naturally not infected by *Wolbachia*, such as *Aedes aegypti (14),(16),(19),(20),(28)* and *Anopheles stephensi (40),(84)*; but also in those that naturally host it, such as *Aedes albopictus (35),(85),(86)*, *Aedes polyniensis (37),(38)*, *Anopheles gambiae (57),(58)*, and *Culex quinquefasciatus (87).* In *Aedine* vector species, transinfected *Wolbachia* blocked the development of several human pathogens: yellow fever virus (YF) *(20)*, chikungunya virus (CHIKV) *(16),(20),(88),(86)* three serotypes of dengue virus (DENV1, DENV2 and DENV3) *(16),(19),(28),(64),(35),(85),(38)*, and the filarial nematode *Brugia pahangi (37*). *Wolbachia*-infected *Aedes aegypti* was also protected against *plasmodium gallinaceum (16)*. In *Anopheline* species, transinfected *Wolbachia* induced protection against *Plasmodium falciparum*, the most virulent plasmodium species *(57),(40)*, modestly suppressed *Plasmodium berghei* oocyst levels *(58)*, and at some temperatures also protected from *Plasmodium yoelii* infection *(84)*. In *Culex pipiens quinquefasciatus,* transinfected *Wolbachia* diminished the *West Nile virus* (WNV) transmission (*87*). Six publications also reported that transinfected *Wolbachia* could enhance infection of some pathogens in vector species such as WNV in *Culex tarsalis (42) and in-vitro (59), Plasmodium yoelii* at some temperatures in *Anopheles stephensi (84)*, *Plasmodium gallinaceum in Aedes fluviatilis (80), Plasmodium relictum in Culex quinquefasciatus (89)*, and DENV2 in *Aedes aegypti (90*). These constitute reversal outcomes, i.e. the reverse to what was intended with an effective *Wolbachia-*based modification.

#### Fitness

None or low fitness costs, caused by the introduction of *Wolbachia* in insects species, allow the modified insect to be reproductively competitive against their natural counterparts, and therefore to easily invade natural populations after its release. Analysed publications described no or low fitness costs after *Wolbachia introduction* in several vector insect species: *Aedes aegypti (67),(22),(24),(73),(30), Aedes albopictus (91),(78),(79),(86), Aedes fluviliatilis (80), Aedes polyniensis (75–77)*, and *Culex pipens quinquefasciatus (81),(82).* Some articles reported a fitness cost that act as part of the control strategy, i.e. constituting part of the modification efficacy. These were the report of *Wolbachia*-induced life shortening of vectors (that reduce or eliminate the time vectors can transmit the pathogen) *(16–19),(68),(34),(57)* or decreased viability of desiccated eggs (preventing the next generation of mosquitoes from hatching after the dry season) *(18),(68).* Despite referring to a fitness cost, since they constitute *Wolbachia* traits that ensure its effectiveness, are herein considered effective outcomes of the Technique topic. In some cases *Wolbachia*-induced fitness costs on mating competitiveness *(41),* fecundity *(22),(27), (80),(34),(41),* fertility *(22)*, larvae competitiveness *(92)*, life span *(34)*, or development time *(68)*,*(25)* of the modified insect. Moreover, some publications reported *Wolbachia*-induced fitness benefits in vector insects *(13),(90),(93),(74),(92),(41),(94),(95),(82).* Once fitness benefits lead to an increase of the insect vectorial capacity and consequently to an increase in disease transmission, they constitute reversal outcomes.

#### Release

A successful release of modified insects describes either (i) their effective invasion and establishment in the field (replacing natural population) or (ii) incompatible mattings between modified and natural insects (suppressing the population). The release of the non-vector *Ceratitis capitata* (fruit fly) males, transinfected and inducing complete CI, led to the complete suppression of a laboratory cage population of natural specimens *(44)*. Insects with introduced *Wolbachia* successfully replaced natural specimens in laboratory cages *(12),(44),* in semi-field cages *(19),(40)*, and in the field, *(67),(27).* Similar results were suggested by computational modelling studies *(96),(30).* Other articles reported invasion but only under certain meterological *(68)* or entomological conditions *(26*), or if some technical ordeals could be overcome *(97)*. However, release of *Wolbachia* insects also led to no/low invasion rates *(98),(99).* Several studies suggested the need to release prohibitively large number of insects *(100–102).* To overcome that, two solutions were reported: releases in a ratio of 95% male mosquitoes (requiring a mass rear capacity) (*11*) or the introduction of insecticide resistance genes along with *Wolbachia* in the host insect, combined with a pre-release intervention to reduce (adult) insect vector numbers *(29)*, *(3)*. The unintended increase of the insect population after the release of the modified insects (reversal outcomes) was estimated by computational modelling studies *(103)*, some of them based on field data of wMelPop-*aegypti (100)* and of superinfected *Aedes albopictus (93)*.

#### Epidemiology

Until 2016, no publications described the impact of *Wolbachia*-based modified insects on human disease incidence. However, several computational modelling studies estimated a successful epidemiological impact after the release of *Wolbachia*-insects *(96),(11)*, specifically using wMel-*aegypti* combination which seems to eliminate DENV transmission in low or moderate transmission settings *(104),(105)*.

#### Long-term

Released wMel-*aegypti* populations persisted in near fixation and maintained the *Wolbachia*-induced DENV protection, two years after the release *(27),(28)*. Laboratory and/or computational modelling studies also revealed that *Wolbachia* (natural or introduced) may persist over time in its symbiotic host *(106),(107),(10),(108).* Despite that, the long-term efficacy of *Wolbachia*-based modification strategy was questioned due to the report of (i) a change in CI rates or in *Wolbachia* density with age, time, or over generations *(109–111),(69),(112),(47),(113-115)*; (ii) a change in other effective traits *(45),(60)*; (iii) loss of *Wolbachia* infection *(106),(116–122)*; and (iv) its natural replacement by other *Wolbachia* strain *(123–125)*. Moreover, long-term efficacy was also questioned by the risk of immigration/re-invasion of other insect populations after the release and fixation of the modified insect population *(21),(27)*. How and how much this phenomena will affect efficacy is not known *(104),(21)*.

#### Variability

Finally, also affecting *Wolbachia* efficacy is its variability. It was reported that a considerable degree of variability may evolve in short evolutionary periods *(126)*. Several articles described *Wolbachia* evolution *(127),(128)*, including its transition from facultative parasite to a nutritional mutualist *(129)* or obligatory symbiont *(130)*. *Wolbachia* density inside an insect-host changed according to a multitude of factors, such as, host genetic background *(112),(131),(61),(132),(133),(62),(134)*, presence of resistance genes *(135)*, host gender *(109),(136),(111),(134)*, development stage *(111)*, nutrition *(57),(41),(137),* immunity status *(111)*, presence of pathogens *(23),(83)*, or host microbiome *(39),(138–140).* It also varied according to *Wolbachia* strain *(141),(142)*, even when coexisting in the same host *(140)*, insect larvae density *(143)*, and environmental conditions *(84),(144),(145),(143),(111)* (such as humidity and temperature). However, *Wolbachia* density in *A. aegypti* did not alter after repeated human blood feeding *(31)*, neither insecticide susceptibility of *A. aegypti* changed after *Wolbachia* infection *(36).* Somewhat surprising *Aedes albopictus* cell lines infected with wStr or wAlbB showed resistance to streptomycin *(63). Wolbachia* main effective outcomes in insect vectors are presented in Table 1 and its reversal outcomes in insect vectors are described in Table 2, see the complete data (also (146–153) and (154–201)) in S1 Table.

**Table 1.** Main effective outcomes of Wolbachia-based and other symbiont-based insect modification (vector insect species).

Species	Wolb strain	Release	Epidemiology	Long-term	Type of Study	Reference
*Aedes aegypti*	wAlbB	reaching infection fixation within seven generations			Lab	Xi et al., 2005a *(11)*
	wMel/ wMelPopCLA	near fixation in 30 days-wMel (much quicker than wMelPopCLA)		wolb-mosquitoes would be successfully maintained in wild populations	Lab and semi field	Walker et al., 2011*(18)*
	wMel	near-fixation in 5 weeks / 90% infected mosquitoes at 5 weeks after releases			Field and Model	Hoffmann et al., 2011 *(66)*
	wMelPop-CLA	invasion is possible under humid conditions *(under dry conditions invasion will be difﬁcult)*			Lab and Model	Yeap et al., 2011 *(67)*
	wMel		can eliminate dengue transmission in low or moderate transmission settings	wolb-mosquitoes once established these are not vulnerable to invasion	Model	Hughes and Britton, 2013 (*103)*
	n.a.	(achieve fixation in a comparable time but with half mosquitoes)		the approach can be used to bolster wolb frequency if reinvasion by uninfected mosquitoes occur.	Model	Hoffmann and Turelli, 2013 *(2)*
	wMel	Residential blocks with relatively low numbers were more easily invaded			Field and Model	Hoffmann et al., 2014a (*25)*
	wMel	near fixation in both locations, *but a persistent low frequency of uninfected mosquitoes*		>2 years after release (traits were reevaluated)	Lab and field	Hoffmann et al., 2014b (*26)*
	wMel			>2 years after release protection persist	Lab and field	Frentiu et al., 2014(*27)*
	n.a.		66-75%reduction in DENV transmission *(it may be insufficient in high transmission settings)*		Model	Ferguson et al., 2015(*104)*
	wMel	can spread effectively in different urban environments			Lab, field and Model	Dutra et al., 2015 (*29)*
*An. stephensi*	wAlbB	invasion of laboratory mosquito populations			Lab and semi field	Bian et al., 2013a *(39)*
*Culex pipiens*		*If technical ordeals can be overcome*, wolb can invade vector populations			Lab and Model	Rasgon and Scott, 2003 *(96)*
*Glossina mors. mors*	modified *Sodalis’*	Fixation of the modified tse tse flies	potential to eradicate trypanosome infections in humans, animal reservoir		Model	Medlock et al., 2013 *(1)*

Note: Publications reporting effective outcomes in release, epidemiology and long-term topics (the ones closer to the strategy aim, that is transmission blockage or vector suppressing). Ineffective outcomes of the mentioned publications are also presented (in italic). Lab stands for laboratorial and Model stands for computational modelling paratrangenesis.

**Table 2.** Reversal outcomes of Wolbachia-based and other symbiont-based insect modification (vector insect species cell-lines not included).

Species	Modification Strategy	Release	Long-term	Type of Study	Reference
*Aedes aegypti*	anti-pathogen Transgenesis (MLA*)	the risk of an accidental premature release into nature is minimized and can be used as a back-up transgene dispersal mechanism *while not as efficient as active drive mechanisms*		Model	Rasgon, 2009 *(152)*
	Lethal Transgenesis (RIDL)	males introduced weekly eliminated the populations within 10–20 weeks.		Lab	Wise de Valdez et al., 2011 *(169)*
	Lethal Transgenesis (RIDL)	substantial suppression can be achieved if releases are deployed in a uniform spatial pattern using strains combining multiple lethal elements		Model	Legros et al., 2012 (*170)*
	Lethal Transgenesis (Female killing)	Release ratio and population size can impact mean extinction time. Eradication may *not always* be obtainable in an operationally realistic time frame		Model	Robert et al., 2013 *(172)*
	anti-pathogen Transgenesis	the most efficient approach for achieving spread of anti-pathogen genes within three years is generally to release adults of both sexes in multiple releases over time. *(the less efficient male-only release impose less public concern)*		Model	Legros et al., 2013 *(163)*
	anti-pathogen Transgenesis	can substantially decrease vector competence of a natural population, even at release ratios well below those required for population reduction. Are considerably more robust to immigration.		Model	Okamoto et al., 2014 *(167)*
	Lethal Transgenesis (Female killing)	can decrease vector competence of a natural population, at release ratios not as low that required for anti-pathogen gene [above].	*are compromised by immigration of wild-type mosquitoes*	Model	Okamoto et al., 2014 *(167)*
*Anopheles stephensi*	anti-pathogen Transgenesis	(in lab cages) gradually replaced non-transgenics mosquitoes when fed on Plasmodium-infected blood *but not when fed on non-infected blood*		Lab and Model	Marrelli et al., 2007 *(150)*
	anti-pathogen Transgenesis	(in lab cages) transgenic mosquitoes invade when maintained on Plasmodium-infected blood.		Lab and Model	Smith, 2013 *(164)*
*Culex pipiens sl*	anti-pathogen Transgenesis	the number of transgenic mosquitoes that must be eventually released may be low and the gene of interest could spread in a relatively short period of time		Lab and Model	Rasgon et al., 2006 *(6)*
*Glossina mo..morsitans*	anti-pathogen Transgenesis	experimental CI results were incorporated into a mathematical model, confirmed that *Wolbachia* can be used successfully as a gene drive		Lab and Model	Alam et al., 2011 *(3)*

Note: Publications reporting reversal outcomes (reverse to the strategy aim, that is transmission blockage or vector suppressing). Effective outcomes of the mentioned publications are also presented (in italic).

*MLA – Multi Locus Assessment; Lab stands for laboratorial and Model stands for computational modelling.

### Transgenesis and other non-symbiont-based modification strategies (effective and ineffective outcomes)

A total of 37 research articles (15.6% out of the 237 with efficacy outcomes) have results regarding the efficacy of transgenesis or other non-transgenic and non-symbiont-based insect modification (37/100% reported effective outcomes, 15/40.5% reported ineffective outcomes).

Two types of transgenesis were found in analysed publications: (i) anti-pathogen transgenesis, i.e. transgenesis with an anti-pathogen effector gene (30 publications*) (202–205),(9),(206),(7),(207), (208),(5),(209–215),(6),(4),(216),(8),(217–225)* and (ii) results on lethal transgenesis, i.e. transgenesis with a lethality inducing gene, covering the release of insects with a dominant (RIDL) (three publications) *(226–228)*, and with a female-killing transgene (three publications) *(229),(224),(230).* Two non-transgenic non-symbiont-based insect modifications were also reported: (i) a radiation-based sterilization insect technique (SIT) (one publication) *(231*), and (ii) a RNAi-mediated sterilization (one publication) *(232*).

**Figure 5.**
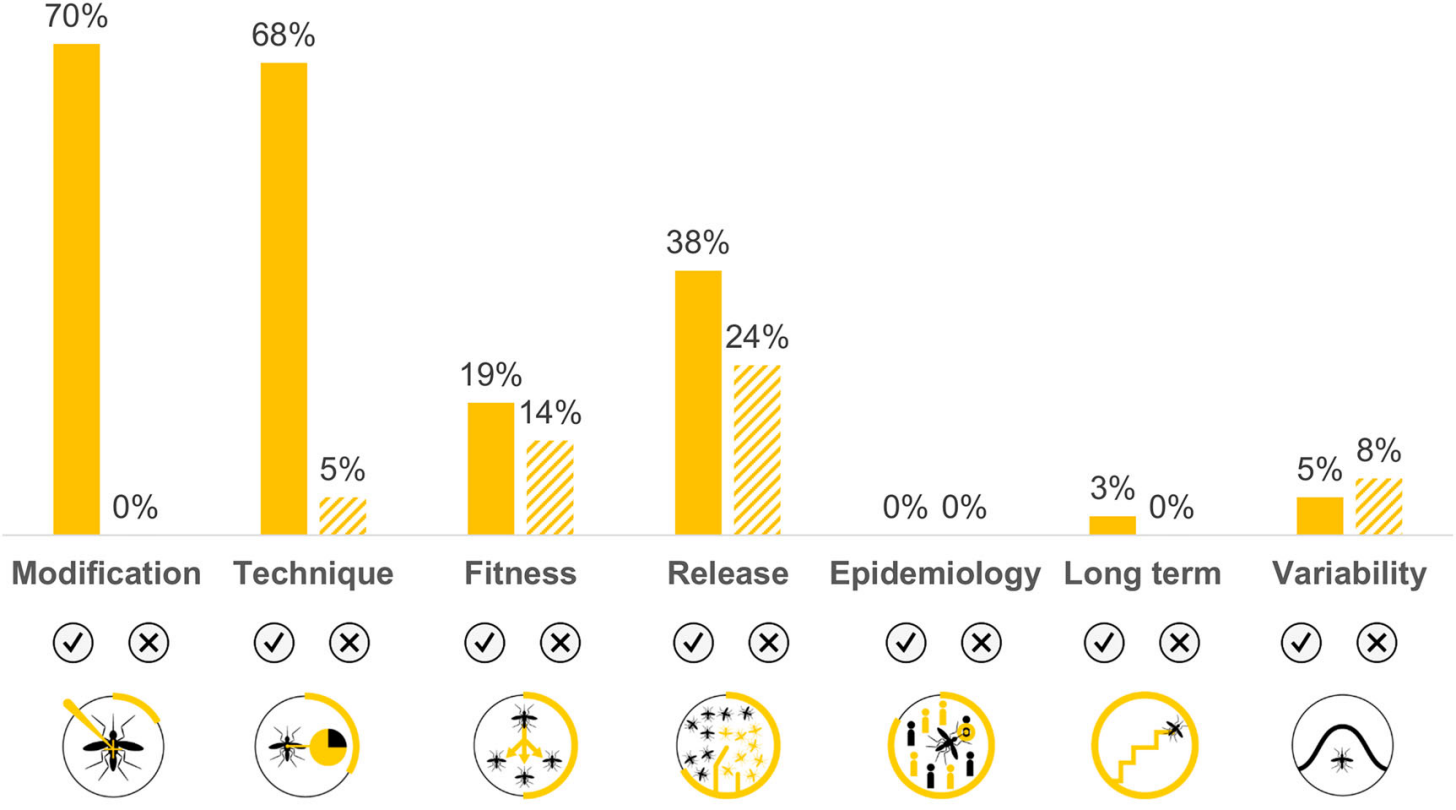
Distribution of the publications regarding Transgenesis and other non-symbiont-based modification strategies, whose results contribute to each efficacy topic, distinguishing effective/ineffective outcomes (*n* = 37).

Not all modifications covered all efficacy topics (Modification, Technique, Fitness, Release, and Variability). No studies reported results regarding epidemiology efficacy using these types of modifications. Results are described in the following paragraphs and quantified in Figure 5.

#### Modification (anti-pathogen transgenesis)

Several studies described successful gene vectors mainly transposable elements, such as *Hermes* and *piggyBac (230),(202),(205),(211),(213),(216),(218),(221),(223),(228)*, but also transcription activator-like effector nuclease (TALEN) (225). Moreover, several distinct promotors were described generating successful sex, tissue or stage-specific expression of effector genes *(211),(212),(204),(213),(203),(217),(219),(215).* Successful insertion of an anti-pathogen effector gene was reported in *Aedes aegypti (203),(208),(211),(212),(216),(220),(217),(224), Anopheles gambiae (204),(213),(218),(222),(205),(207),(210),(221), Anopheles stephensi (205),(207),(210),(214),(221), Culex pipiens (7*), G*lossina morsitans morsitans (4)*, and in some non-vector insects *(202),(9),(6),(215),(8),(219),(223),(225)*.

#### Technique (anti-pathogen transgenesis)

A subsequent blockage or reduction of pathogen transmission was reported in *Aedes aegypti (208),(211),(212),* in *Anopheles gambiae (204),(213),(222), Anopheles stephensi (207),(214),(221)*, and in the silkworm *Bombyx mori (225)*. However, in one publication the *Plasmodium falciparum* protection induced by an anti-malarial gene was inconsistent *(213)*.

#### Fitness (anti-pathogen transgenesis)

In some cases, the anti-pathogen transgene led to no or low fitness cost in the modified insects *(208),(222),* thus allowing the modified insect to be reproductively competitive against their natural counterparts. However, anti-pathogen gene insertion also caused fitness benefits in *Anopheles gambiae* (*206)*, and *Anopheles stephensi* that fed *Plasmodium*-infected blood *(207),(221).* Since these outcomes increase vectors abundances and vectorial capacity, they constitute reversal outcomes.

#### Release (anti-pathogen transgenesis)

Several publications, all of them computational modelling or laboratorial studies, estimated successful release of insects modified with an anti-pathogen transgene *(7),(207),(4),(220),(221),(224).* Some out of those described the efficacy of different gene drives (which bias the inheritance of a particular gene to quickly and irreversibly spread it through a population) such as, Multi-locus assortment *(209)*, Medea and Killer-Rescue *(220*), and *Wolbachia (7),(4),(9),(5).* Two publications presented comparative studies describing advantages and disadvantages of several gene drives *(6),(8)*. Numerous computational modelling studies suggested a hard compromise between invasiveness and confinement (a high migration rate required to become established in neighbouring populations, and low frequency persistence in neighbouring populations for moderate migration rates) *(209),(220),(7),(4),(9),(5),(6),(8). Wolbachia* was referred as an efficient gene drive in some studies *(9),(7),(5),(4)* but according to Marshal and Hay, 2012 *(8)*, was not reliable for confinement properties. Semele, Merea and two-locus engineered under-dominance were the most promising in conﬁnement properties and required lower introduction frequencies (compared to *Wolbachia*, Medea, single-allele under-dominance, single-locus engineered under-dominance, and killer-rescue) *(8)*. Multi Locus Assessment, despite being less effective as gene drive, allows the test of ecological components before releases with more invasive gene drives *(209)*.

#### Modification and Technique (lethal transgenesis)

Successful insertion of a lethal transgene was reported in *Aedes aegypti (226), Drosophila melanogaster (230)*, and *Ceratitis capitata (228)* and subsequent lethality was laboratorial confirmed in *Aedes aegypti (226)* and *Drosophila melanogaster (230)*.

#### Fitness (lethal transgenesis)

One publication reported no fitness costs caused by the modification on the modified insect and confirmed that the lethal transgene did not affect insecticide susceptibility *(228)*.

#### Release (lethal transgenesis)

Computational modelling studies reported that the elimination of vector insects might be an unrealistic objective. However, substantial suppression can nonetheless be achieved in certain conditions, such as a uniform spatial pattern and multiple lethal elements *(227)*, or a certain release ratio and population size *(229),(224)*. Elimination of a natural population after the release of insects with a dominant lethal was though reported in a semi-field study *(228)*.

#### Long-term (lethal transgenesis)

One computational modelling study suggested lethal transgenesis long-term efficacy be compromised by invasion of wild type insects *(224)*.

From all publications in this section, only one publication reported a field study, describing the ability to mate and copulate of a radiated insect, modified by a sterilizing technique (SIT) *(231)*. Also only three publications reported reversal outcomes, related to fitness benefits (as above mentioned). Main effective outcomes of transgenic and other non-symbiont-based modified insect vectors are presented in Table 3 (the complete data is presented in S2 Table).

**Table 3.** Main effective outcomes of transgenesis and other non-symbiont-based insect modification (vector insect species).

Species	Wolb strain	(Modification)	Technique (Pathogen protection)	Fitness	Release	Type of Study	Reference
*Aedes aegypti*	wAlbA and wAlbB	Stable transinfection		higher fecundity females		Lab and Model	Ruang-areerate and Kittayapong, 2006 (*13*)
	wMelPop	not applicable			net increase in mosquito numbers may occur	Lab and Model	Jeffery et al., 2009 (*100*)
	wAlbB	Stable transinfection	*(inhibit DENV2 infection)*	increased longevity		Lab	Bian et al., 2010 (*90*)
*Aedes albopictus*	wAlbA & wAlbB	(natural)		live longer, produce more eggs, and have higher hatching rates	eventual undesirable increase in the density of adult population	Lab, semi field and Model	Dobson et al., 2002 (*93*)
	wAlbA and wAlbB	natural, introgressed		longer lived, higher egg hatch in compatible crosses, and more fecund		Lab	Dobson et al., 2004 (*74*)
				under low competitive pressures, females experience higher survivorship		Lab	Gavotte et al., 2010 (*92*)
*Aedes fluviatilis*	wFlu	(natural)	enhances oocyst infection of *plasmodium gallicaceum*			Lab	Baton et al., 2013 (*80*)
*An. gambiae*	wAlbB	somatic infection	increases *plasmodium berghei* oocysts			Lab	Hughes et al., 2012a (*58*)
*An. stephensi*	wAlbB	Somatic transinfection	*plamodium yoelii* (increased oocysts at 24°C)			Lab	Murdock et al., 2014 (*84*)
	wAlbB			Increased life span (sugar meals), despite reduced fecundity		Lab	Joshi et al., 2014 (*41*)
*Culex pipiens*	wPip			protection from *Plasmodium*-induced mortality		Lab	Zélé et al., 2012 (*95*)
	wPip (ARwp line)		increasing *plasmodium relictum* transmission stages			Lab	Zélé et al., 2014 (*89*)
*Culex quinquefasciatus*	wPip	(natural)		live longer, lay eggs earlier and higher hatching rates		Lab	Almeida et al., 2011 (*94*)
*Culex tarsalis*			enhanced WNV infection (not transmission)			Lab	Dodson et al., 2014 (*42*)

Note: Publications reporting effective outcomes on the topics: release, epidemiology and long-term (the ones closer to the strategy’s aim, that is transmission blockage or vector suppressing). Ineffective outcomes of the mentioned publications are also presented (in italic). Lab stands for laboratorial, Model stands for computational modelling, and NA stands for not applicable.

Five articles (2.1% out of the 237 with efficacy outcomes) contributed to the efficacy of insect modification as a vector control approach, regardless of the modification strategy used (see S8 Table). They reported results as diverse as: effective releases, in what concerns numbers of insects, and sex ratio, (233), the impact of laboratory rearing (234), or descriptions of gene flow of eventual release sites (235–237). Interestingly, in all analysed publications regarding gene flow, no isolation was found between Islands and mainland, neither in Society Islands of French Polynesia (236), nor Lake Victoria in Western Kenya (235), nor in Bijagós archipelago in Guiné-Bissau (237).

## Effects

**Figure 6.**
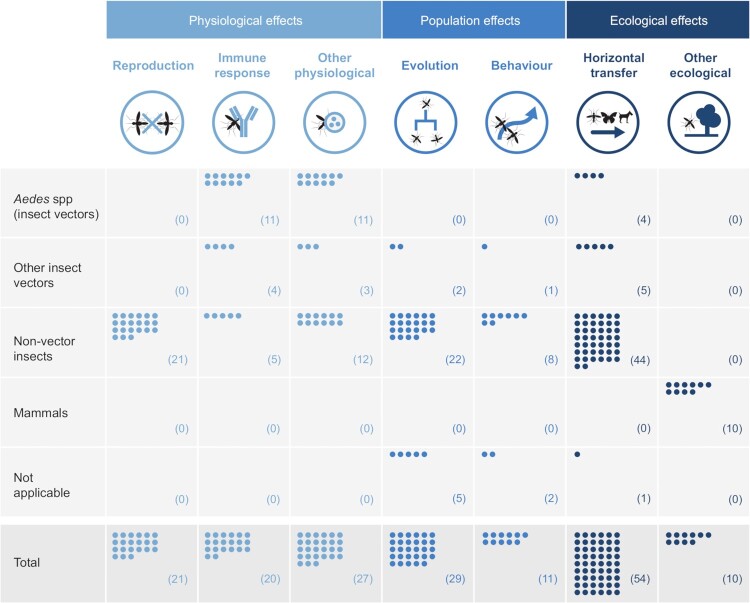
Number of publications reporting each Wolbachia-induced effect per taxonomic group.

Apart from the intended effective traits, modifications also induce other effects into the insects, as reported in 155/44.4% publications (nTotal = 349). Modifications induced effects at several levels: (i) at the specimen level, i.e. physiological effects such as reproduction, immune response, or microbiome of the modified insect; (ii) at the insect species level, concerning its evolution and/or behaviour; and (iii) at the ecosystem level, affecting any other organism of the modified insect ecosystem (Figures 2 and 6).

Majority of publications contributing to this theme covered effects specifically induced by *Wolbachia and* as a natural infection. There were two exceptions only, all of them reporting population effects: one publication describing effects on evolution, induced by other symbiont (natural infection of *Rickestia*) *(1*), and one publication describing loss of assortative mating induced by laboratory rearing *(234)*.

### Physiological effects (at the insect specimen level)

A total of 63/18.1% publications (nTotal = 349) reported that insect modification strategies may induce physiological effects on the target insect. The most frequent physiological effects found in analysed publications were *Wolbachia*-induced effects on reproduction. The following reproductive modifications were reported: (i) male-killing (the death of male embryos during early embryonic development, with advantage for the surviving infected female siblings), stated in four non-vector species (*Ephestia Kueheniella –* butterfly, *Hypolimnas bolina-* butterfly, *Ostrinia scapulalis moth*, and *Tribolium madens – beetle*) *(53),(54),(155),(238–240)*; (ii) feminization (the conversion of genetic males into functional females), described in two non-vector species (*Eurema hecabe* and *Zyginidia pullula*) *(241–243),(172)*; and (iii) parthenogenesis (the exclusive participation of females on reproduction, and production of female offspring), reported in several species of parasitoid wasps *(173),(244),(245).* Although cytoplasmic incompatibility (CI) can be considered a *Wolbachia* reproductive effect, since it is a required trait for *Wolbachia* use as control strategy, it was herein considered an efficacy trait rather than a reproductive effect (see CI results on Technique topic). Moreover, effects on reproduction also included sex ratio alterations *(246),(157),(247),(248)*, exceptional sex mosaics *(168)*, requirement of *Wolbachia* for oogenesis *(130),(131)*, or changes in expression of genes associated with reproduction *(249),(172)*.

*Wolbachia* also induced effects on the immunity of the modified insect mainly through the up-regulation of effector genes. Report of *Wolbachia*-mediated induction of immune system was described in *Aedes aegypti (14),(90),(250),(187),(251),(23)*, *Aedes albopictus (252),(253),(35), Aedes polyniensis (37)*, *Anopheles gambiae (254),(253),(57)*, *Anopheles stephensi (40)*, and *Drosophila melanogaster (250),(249). Wolbachia* also induced reduction of the immune response by decreasing the ability to encapsulate parasitoid eggs in *Drosophila simulans (161) or* decreasing the ability to produce lead peroxides *(255)*.

Other physiological effects were also reported, such as, alteration in the insect’s microbiome, *(164),(138),(150),(39),(256)*, gene expression (genes, microRNA, sRNA, or epigenetic effects), *(172),(257),(249),(258–260)*, or in its nutrition and metabolic mechanism *(261),(170),(71),(262–265),(129),(249),(266),(267),(137),(195)*, see all information regarding physiological effects (also *(268),(269),(182),(270),(189),(271)*) in S4 Table.

### Populational effects (at insect population level)

According to 40/11.5% analysed articles (nTotal = 349), *Wolbachia* also affected its host population in several ways such as altering its mitochondrial DNA (mtDNA) pattern, interfering in speciation process, on its behaviour ecology, or others. Changes in mtDNA patterns were reported in several non-vector insects (*272–283*), and in the vector mosquito *Culex pipiens (7).* Phylogenetic analysis of *Culex pipiens* complex populations from three continents indicated a *Wolbachia*-induced drastic reduction of mitochondrial variability, thus profoundly interfering in its population structure *(7)*. Several articles suggested that *Wolbachia* induced speciation, altering genomic diversity *(284)*, leading to premating behavioural isolation *(285),(286)* or to reproductive divergence (due to variable phenotypic effects) (*10*). *Wolbachia*-induced behavioural isolation is more likely in diploid and haploid than in haplodiploids hosts *(287)*, and can be more evident in hybrid zones (*288*). Examples of behavioural changes, all of them reported in non-vector insects or suggested by computational modelling studies, are the sex-role inversion on reproductive ritual *(246)*, the increase of sexual promiscuity *(159),(289)*, and the irreversible loss of sexual reproduction *(290),(173),(291)*.

Symbiont-induced speciation was also reported in *Neochrysocharis formosa* infected with *Rickestia* (*1)*. All data regarding populational effects (also *(292),(293),(160),(170),(247),(294), (172),(295),(144),(296–299))* is presented in S5 Table.

### Ecological effects (at insect ecosystem level)

Finally, 64/18.3% publications reported that *Wolbachia* is also able to induce alterations in other organism rather than its host, interfering thus, with the host ecosystem. The majority of the publications covering this type of effect described the report or the estimation of horizontal transfer events (i.e. transfer between neighbouring contemporary species) of genetic material, such as a gene or a symbiont. Horizontal transfers (HT) can occur through bacteriophages, parasitoids, hemolymph, etc. Reported HT events comprise innumerous type of transfers such as between symbionts *(300),(292),(301), (302–304),(137)*, from *Wolbachia* to insect vector *(305–309)*, from *Wolbachia* to nematode species (*305)*, from insect to insect *(155),(116),(310–312),(118),(313),(294),(107),(314),(248),(315–320),(296),(321–324),(201)*, and from insect to non-insect species *(325),(326)*, insect to bacteria (*327)*. All data regarding HT (also *(328),(329),(119),(167),(305),(330),(122),(331),(302),(245),(325),(332),(333),(307),(334–339)*) are described in S6 Table.

Apart from HT, *Wolbachia* can reach other organisms also inducing ecological effects. All publications reporting non-HT ecological effects consisted of studies in mammals. Those had contact with *Wolbachia* mainly *via* filarial infection. A *Wolbachia*-infected filarial nematode induced or exacerbates filarial diseases pathogenesis such as human subcutaneous dirofilariasis (*340)*, onchocerciasis (river blindness) *(341),(342)*, and lymphatic filariasis *(343),(344)* (Table 4).

**Table 4.** Other ecological effects: publications reporting Wolbachia-induced effects on mammals and respective main results.

	Wolbachia Origin	Results	Analysed Mammal cells	Related disease	1st author, year
Catle (cows)	via Nematode Pathogen *(Onchocerca armillata)*	Presence of *Wolbachia* confirmed in aorta sections from different *Onchocerca Armilla*-infected animals	tissue sections of infected animals	Onchocerciasis (river blindness)	Neary *et al*., 2011 *(345)*
Cats	via Nematode Pathogen (*Dirofilaria immitis*)	*Dirofilaria immitis* infection leads to an immune response against *Wolbachia* proteins	Sera cats and owners’ cats	heartworm disease *(cats/dogs)*	Bazzocchi *et al.,* 2000(*346)*
	via Nematode Pathogen (*Dirofilaria immitis*)	*Wolbachia*-induced a greater acute inﬂammatory response worsening the broncho-reactivity	infected and non-infected breathing patterns	heartworm-associated respiratory disease*(cats/dogs)*	García-Guasch et al., 2013*(347)*
Humans	via Nematode Pathogen (Wuchereria bancrofti)	anti-*Wolbachia* surface protein antibody responses are associated with the presence of chronic filarial morbidity	human serum samples	Lymphatic filariasis	Punkosdy *et al.,* 2003*(344)*
	via Nematode Pathogen (Dirofilaria repens)	specific immune response to *Wolbachia* in patients; *Wolbachia* may indeed participate in granuloma formation	human skin nodules	Human subcutaneous dirofilariasis	Grandi et al., 2008 *(298)*
	via Nematode Pathogen *(Wuchereria bancrofti)*	*Wolbachia* surface protein may also contribute to the suppression of immune responses seen in filarial patients	human patients blood	Lymphatic filariasis	Shiny *et al*., 2012 (*348)*
	(artificially introduced) neutrophil cell lines with *Wolbachia* lipopolysaccharides (LPS)	*Wolbachia* is a major contributing factor in the development of chronic pathology in humans	blood neutrophils from adult healthy volunteers	Onchocerciasis (river blindness)	Tamarozzi *et al*., 2014*(300)*
	unknown	twice detection in 5days of *Wolbachia* genes in human patient with an unknown infection. Later detection of a non-Hodgkin’s lymphoma	blood of a patient with apparent viral infection symptoms	non-Hodgkin’s lymphoma	Chen *et al*., 2014 *(349)*
Mice	via Nematode Pathogen (*Brugia malayi*)	*Wolbachia*-mediated neutrophil activation is an important mechanism to visual impairment and eventual blindness in ocular onchocerciasis	mouse cornea and peritoneal cavity neutrophils	Onchocerciasis (river blindness)	Gillette-Ferguson et al., 2004*(299)*
Cell lines	(artificially infected) (W*olbachia* from *Brugia malayi*)?	*Wolbachia* lipopolysaccharides (LPS) may be one of the major mediators of inflammatory pathogenesis in filarial nematode disease	murine macrophage and mosquito cultures	Lymphatic filariasis	Taylor *et al.,* 2000 (*301)*

Note: Publications reporting effective outcomes on the topics: release, epidemiology and long-term (the ones closer to the strategy’s aim, that is transmission blockage or vector suppressing). Ineffective outcomes of the mentioned publications are also presented (in italic).

## Discussion

To our knowledge, the present work constitutes a unique review on modified insects for vector-borne disease prevention. Rather than being the perspective of an author or a summary of the authors’ selection of publications, this review followed a structured methodological procedure. Furthermore, authors from the present review are not involved in scientific projects to modify insects having, thus, no conflict of interest in the outcomes of this reflection. Moreover, the present review enclosed several types of modifications in any vector or non-vector insect species, covering an uncommon comprehensiveness, and thus, offering an exceptional opportunity to observe trends and to outline the big picture of the modified insects. Non-vector insects were included since most studies regarding techniques are first performed in model insects, such as *Drosophila spp*., before being applied on vector insects. Also, it goes beyond modifications’ efficacy, most commonly covered in current reviews [14,15], exploring research articles also related to modifications’ non-target effects and even the variability of the efficacy outcomes in different settings. Finally, it is to our knowledge the sole review on the subject including publications on mammals and exploring the eventual non-target effects of insects’ modifications on this important taxonomic group.

Efficacy of the modifications was the most covered theme, being analysed in 237 out of the 349 publications included in the review. Nevertheless, the amount of publications regarding modifications’ efficacy does not reflect the extent either the robustness of the evidence available regarding it. Out of those 237, only 41 publications reported main effective outcomes: successful releases of modified insects, its positive epidemiological impact and/or its long-term efficacy (the remaining 196 cover ineffective or primary effective outcomes). Out of the three main efficacy topics, the epidemiological impact was the less covered, reported in only five publications, none of them based on the most robust field studies, but all based on computational modelling studies instead. In fact, results based on modelling results may not be the same when tested in the field (since no modelling study consider all variables of a real environment). Also, results on the release of the modified insects may not persist in the long-term neither mean an actual change on disease transmission.

According to the epidemiology definition, efficacy determines whether an intervention produces the expected result under ideal circumstances, while effectiveness measures the degree of beneficial effect under actual settings [16]. This works explored efficacy (modification, technique, and fitness) and effectiveness-related topics (release, epidemiology, long-term and variability). However, to simplify classification, “efficacy” was used as a broad term that included all above-mentioned topics.

Main effective outcomes were obtained for several insect modifications, namely, *Wolbachia*-based strategies (replacement and suppression approaches), RIDL, and female killing lethal transgenesis (both suppression approaches), computational modelled transgenesis regardless of the transgene (replacement and/or suppression approaches), and paratransgenesis (replacement approach). Seven publications (out of the 41) reported main effective outcomes based on semi-field or field studies (corresponding to outcomes obtained with *Wolbachia* or RIDL strategies). Even though not many, these publications achieved critical outcomes such as (i) field-released *wMel*-*aegypti* mosquitoes not only reached near fixation (despite a persistent low frequency of uninfected mosquitoes), but also maintained their effective traits such as CI, fixation and pathogen protection for at least two years after the release *(27)(28)* (from S1 Appendix); (ii) weekly introduction of *Aedes albopictus* males with a dominant lethal (RIDL) led to the eradication of a laboratory cages population in 10–20 weeks *(228)* (from S1 Appendix).

The present review also described reversal outcomes obtained after the release of transgenic insects or insects modified with *Wolbachia* (reported in 39 publications). Out of those, only one publication corresponds to a field study, but in this case, the release of modified insects may lead to an increase in the vector insect population, particularly if occurring when its natural abundance is at its maximum [17] (also (*100*) from S1 Appendix).

No publication was found directly reporting the non-target effects of modification strategies. Analysed publications reported biological effects (at physiological, populational, and ecological level) of natural *Wolbachia* suggesting eventual non-target effects of *Wolbachia*-based insect modifications (no publications reporting eventual effects of transgenesis and other non-symbiont modifications were found the systematic review until March 2016). The effects on mammals should be particularly and carefully explored. Monitoring of an eventual increase of lymphatic filariasis severity, non-Hodgkin’s lymphoma incidence, or unrecognized infections may be advised in areas already subjected to *Wolbachia*-insects releases. Since the inclusion criteria were restricted to studies on insects and mammals, results regarding ecological effects may be limited. Horizontal transfer events of *Wolbachia* genetic material to non-insect non-mammal species were even though reported *(305),(325),(326*) (from S1 Appendix).

The *Wolbachia* modification technique was the most present in this review. These results do not reflect the actual amount of studies of some techniques such as RIDL or SIT. *Wolbachia* is a natural bacterium, and thus *Wolbachia*-based strategies are much more accessible for study. Regarding transgenic insects, modified strains are patented and often are published with a code name such as OX3604C that escapes to the search expression. Moreover, besides those studies specifically oriented for a *Wolbachia*-based strategy, other studies regarding *Wolbachia* were included (mainly regarding natural *Wolbachia*) whenever their results contributed to the efficacy or the effects of *Wolbachia* as a vector control strategy. Furthermore, *Wolbachia* is a unique term while for transgenesis each strategy may try a different transgene, with a particular effect originating several diverse terms that may not all be covered in research expressions. Also, regarding radiation-based modifications, such as SIT, some publications may have been excluded during 1st screening since their efficacy results concern studies of the modified insect for agriculture purposes (and not for disease prevention). Due to the high number of articles found initially (1204) the application of exclusion criteria was critical to turn the analysis feasible.

Even though there were not found publications reporting eventual effects of transgenesis and other non-symbiont modifications, some of their effects were already discussed in the literature. In what concerns suppressing strategies, such as RIDL (lethal transgenesis), female killing (lethal transgenesis), SIT, or RNAi-mediated sterilization, the elimination of a species leads to profound changes in its ecosystem, such as eventually putting some non-target species in risk or giving opportunity to not-targeting species to expand [18,19]. Moreover, some authors have been arguing that biodiversity loss may even be associated with emergence of vector-borne diseases [20,21]. Regarding replacement approaches, such as anti-pathogen transgenesis, the effects of the transgene in the ecosystem are unknown. When associated with *Wolbachia* as gene drive, there are emerging questions regarding the lateral transfer of the inserted transgene to *Wolbachia* itself or via *Wolbachia* to other organisms.

In what concerns the year of the publications it is clear how recent this topic is, being almost the totality been published in the last 20 years. This can, at least partially, explain why in several topics we found a gap of knowledge, such as the long-term efficacy or the epidemiological impact of the modifications.

This hot topic is the subject of several publications each month. An equivalent search applying the same Mesh Terms founds more than 1250 articles that have been published since March 2016 until 2019. Thus, systematic reviews of proliferous subjects as the present one are, by definition, always delayed regarding current knowledge. However, they are of paramount importance since they constitute a baseline of knowledge that not only point out to questions that may need to be further explored, but also can be fed with forthcoming publications to follow up trends. Moreover, the present review developed a framework of themes, topics and outcomes, offering an analysis scheme that may facilitate future updates. Evidence to support these conclusions can be found when exploring recent important articles. We selected (non-systematically) 12 relevant articles published since March 2016 until now [22–32]. Critical successful evidence was reported, mainly field-based: (i) first stable transgenic CRISPR/cas9 *aegypti-*mosquitoes strain [23]; (ii) transgenic *Anopheles* population collapse in semifield using CRISPR/Cas9 [19]; (iii) near elimination of field mosquitoes after release of irradiated and *Wolbachia*-infected mosquitoes [24]; (iv) higher virus blockage of *Wolbachia*-infected field-reared mosquitoes [25]; (v) *wolbachia*-mediated pathogen inhibition for ZIKV and ZIKV/DENV co-infection [26]; and (vi) released *Wolbachia*-aegypti stable over 12 months and 28 months at some sites, and reduced human dengue incidence [27,28]. However, the most needed effective epidemiologic and long-term outcomes are still absent or scarce. On the contrary, recent articles reinforced questions regarding long-term efficacy with reports of post-release re-infestation of wild type mosquitoes [29], and post-release loss of *Wolbachia* from modified mosquitoes [30]. Recent critical evidence also comprise the detection of *wolbachia* in *A. aegypti* [31], and the successful field establishment of pyrethroid-resistant modified mosquitoes [22], and the surprising generation of hybrid mosquito after sterile mosquito release [32]. These findings reinforce the complexity implicit on modifying preventive strategies and the eventuality of unpredictable and irreversible effects. Even though these articles were not included in the systematic review, their main findings are presented in Box 1, following the same analysis scheme of themes, topics, and outcomes presented in Figure 1.

**Box 1.** Top 12 articles covering critical evidence regarding modified mosquitoes for disease prevention, published after March 2016 (non-systematic selection of articles). Main findings are presented following the same analysis scheme used in the present systematic review.

		Efficacy			(Non-target) Effects				
Species	Modifying technique	Modification/Technique/Fitness	Release/Epidemiology	Long-Term/ Variability	Physiological	Population	Ecological	Type of Study	Reference
*Aedes aegypti*	Transgenesis (RIDL)			Re-infestation by wild type mosquitoes 4–5 months after the release.				Field	Garziera et al., 2017 [27]
	Other transgenesis (CRISPR/Cas9)	Generation of multiple stable, transgenic mosquito strains expressing Cas9 in the germline						Lab	Li et al., 2017 [22]
	*Wolbachia*		*wAlB-*mosquitoes successfully established and reduced human dengue incidence was registred	*wAlB* frequencies remained high and stable at some sites				Field	Nazni et al., 2019 [31]
	*Wolbachia*		*wMel-*mosquitoes successfully established across 66 km^2^ and no local dengue transmission was registred	Establishment persisted over 28 months				Field	O’Neill et al., 2019 [32]
*Anopheles gambiae*	Other transgenesis (CRISPR/Cas9)		Reaching 100% prevalence within 7–11 generations leading to total population collapse					Semi field	Kyrou et al., 2018 [23]
*Aedes aegypti*	*Wolbachia*		The field-reared mosquitoes have greater *wMel*-mediated inhibition of DENV infection than their lab-reared counterparts.					Lab and Field	Carrington et al., 2017 [25]
	*Wolbachia*			*wMel-*mosquitoes with pyrethroid resistant genetic background have successfully established, despite fitness cost (contrary to their susceptible counterparts)			(also affecting here)	Lab and Field	Garcia et al., 2019 [21]
*Aedes aegypti*	*Wolbachia*	ZIKV and ZIKV/DENV co-infection transmission blockage						Lab and Field	Caragata and Rocha, 2019 [26]
	*Wolbachia*			Detection of natural/native *Wolbachia* in Ae. *aegypti*				Field	Carvajal et al., 2019 [29]
	Transgenesis (RIDL)		Rare viable offspring was produced from sterile transgenic male, generating new hybrid mosquito population				(also affecting here)	Lab and Field	Evans et al., 2019 [30]
	*Wolbachia*			Decline and loss of *Wolbachia* density from *wolb*-modified mosquitoes in field				Lab and Field	Ross et al., 2019 [28]
*Aedes albopictus*	SIT and *Wolbachia*		Radiation- and wolb-based modified mosquitoes almost eliminate field mosquito population					Field	Zheng et al., 2019 [24]

In conclusion, insect-modification strategies appear as a promising innovative alternative to overcome an unprecedented increase of vector-borne, mainly arboviral, diseases. Nevertheless, these modification tools still lack field evidence, mainly regarding epidemiological and long-term efficacy.

Field releases in endemic areas could hopefully provide that kind of missing epidemiologic and long-term evidence. However, unintended effects such as reversal outcomes on disease transmission, or irreversible biological effects (including effects on mammals) need to be explored, dispelled, or resolved. This leads to demand for studies before open release assays. Semi-field evidence may have a relevant role in this impasse. Since we could only have a robust knowledge, if these strategies would be implemented, health authorities should reflect to what extent and in which circumstances the risk is worth value.

The level of variability of existing evidence suggests the need to generate local/specific evidence in each setting of an eventual release. Importantly, available preventive strategies should not remain on hold while modified insects do not offer an effective and safe solution. This reflects the huge dilemma that is under the use of modified insects to prevent vector-borne diseases. A comprehensive cost-effectiveness analysis could be an important tool when deciding to proceed or not with these innovative strategies, and/or to improve traditional available strategies.

## Supplementary Material

Supplemental Material
