# Supplementary material for: What happens when we modify mosquitoes for disease prevention? A systematic review
Source: Emerg Microbes Infect. 2020 Feb 11;9(1):348–65. doi: 10.1080/22221751.2020.1722035 (PMC7034073; doi:10.1080/22221751.2020.1722035)

S1 Fig - Quantitative description of the included publications according to: type of insect modification strategy (A), type of study (B), study's species (C), year of publication (D)

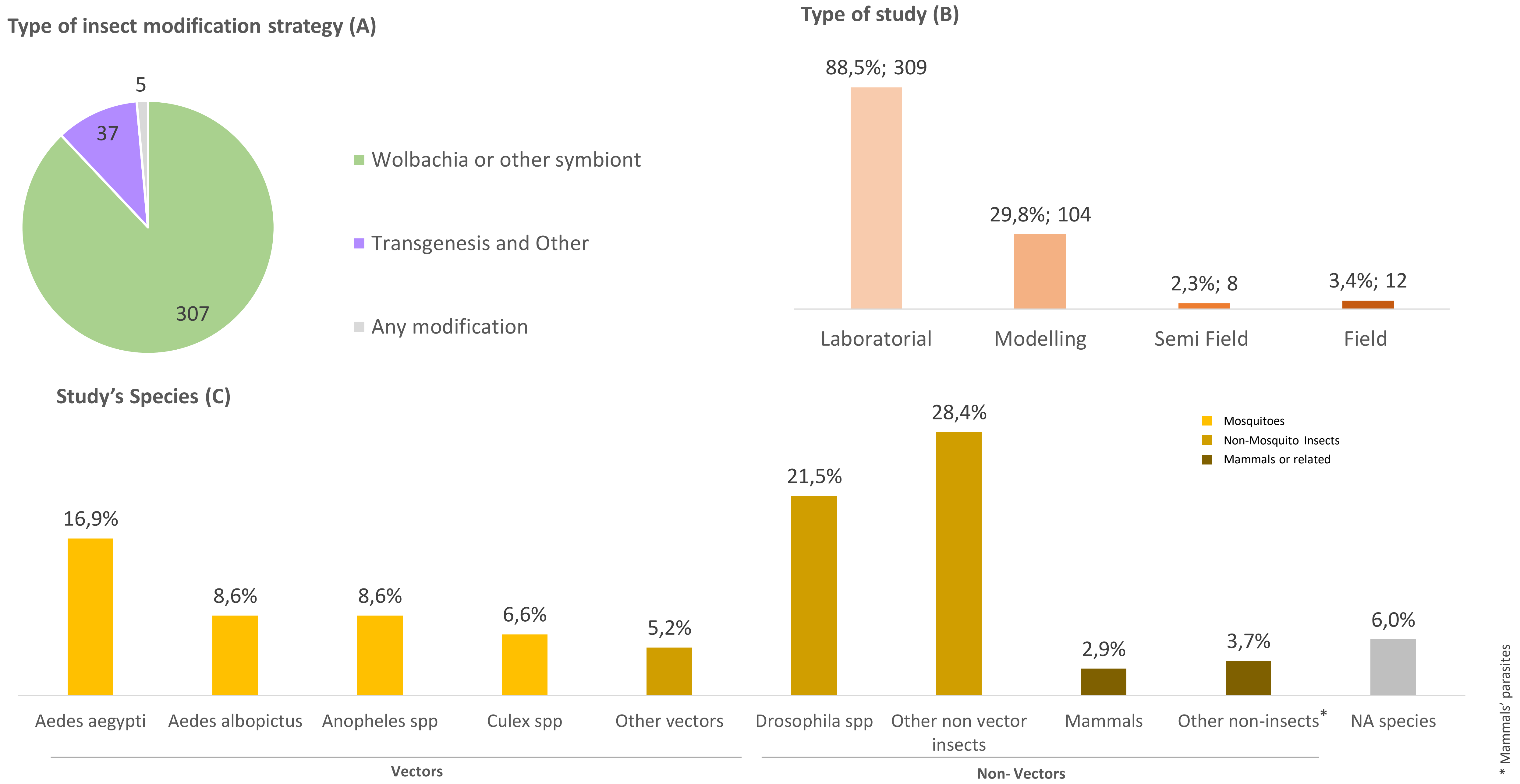

**Year of publication (D)**

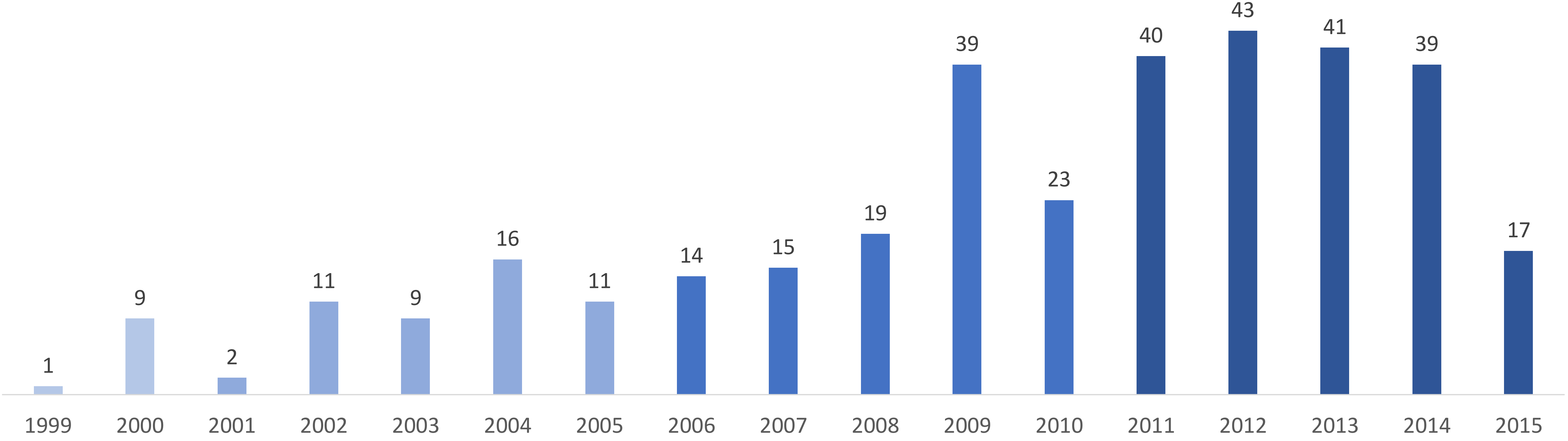


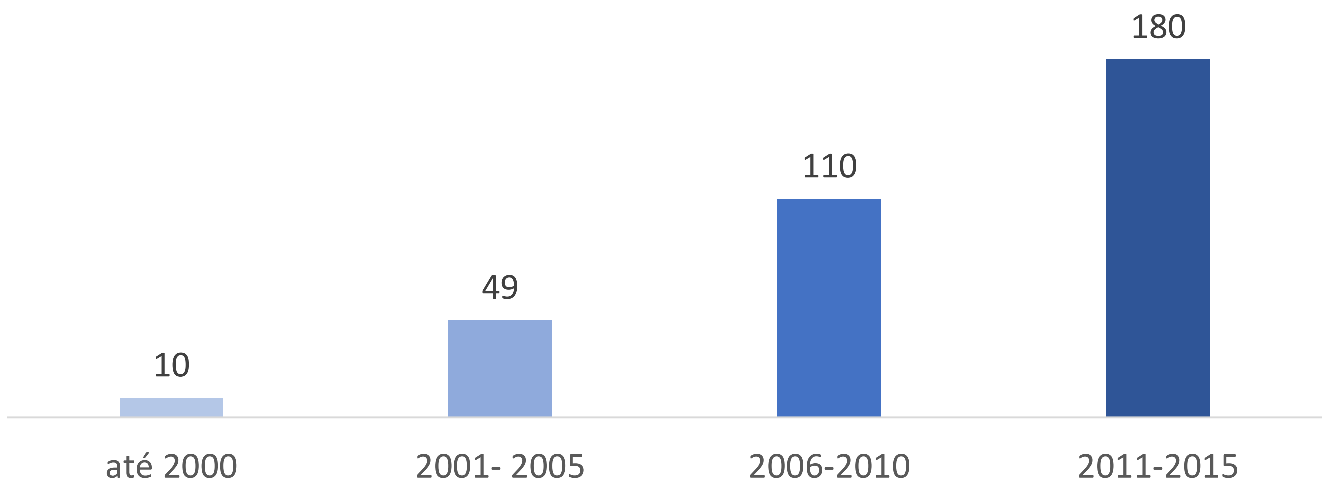

Supplement: Supplemental Material [file TEMI_A_1722035_SM1666.zip › Supplementary Material/S1_Fig.pdf]
