# Supplementary figures and images for: What happens when we modify mosquitoes for disease prevention? A systematic review
Source: Emerg Microbes Infect. 2020 Feb 11;9(1):348–65. doi: 10.1080/22221751.2020.1722035 (PMC7034073; doi:10.1080/22221751.2020.1722035)

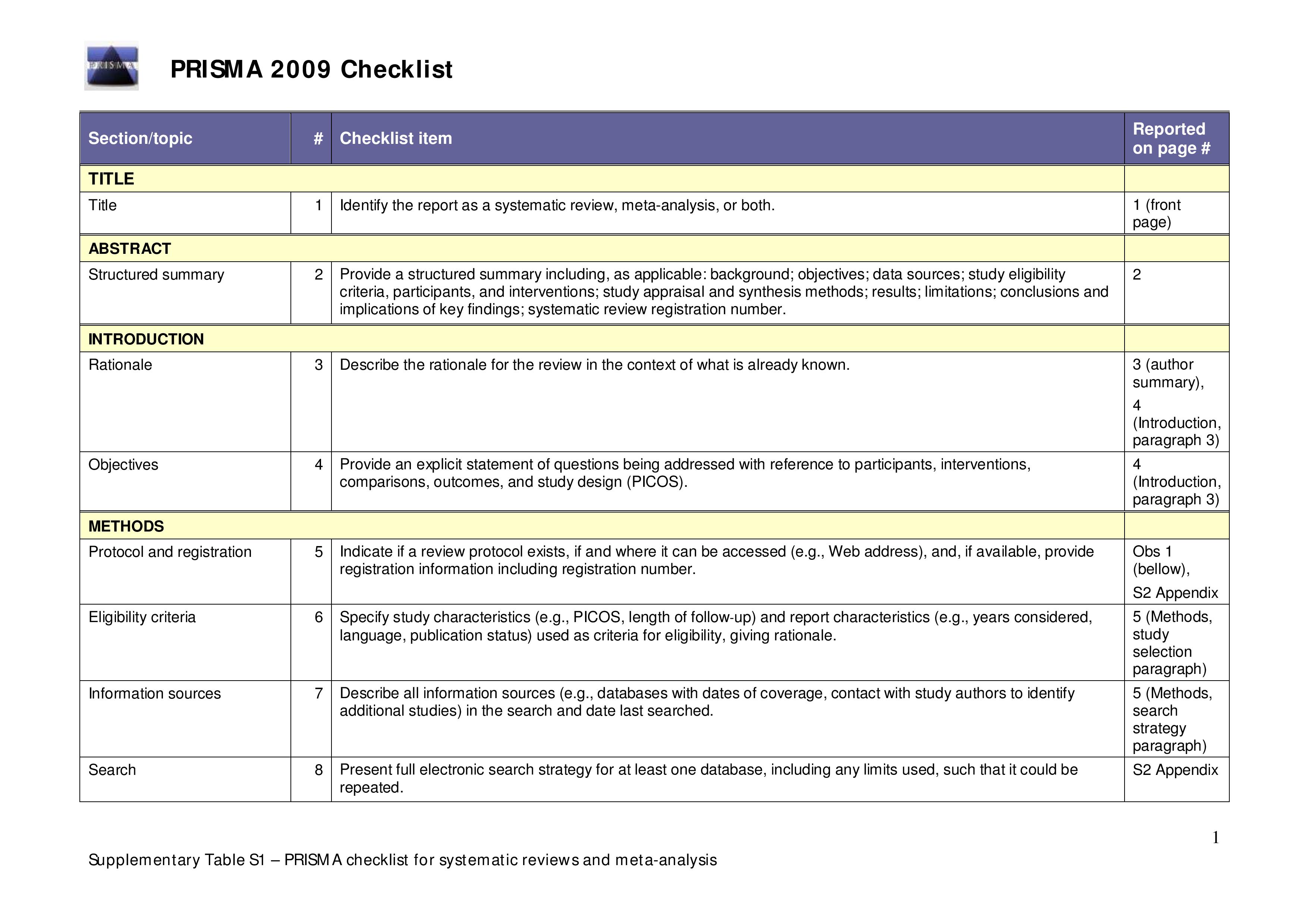

Supplement: Supplemental Material [file TEMI_A_1722035_SM1666.zip › Supplementary Material/S1_Checklist_page_001_final.jpg]

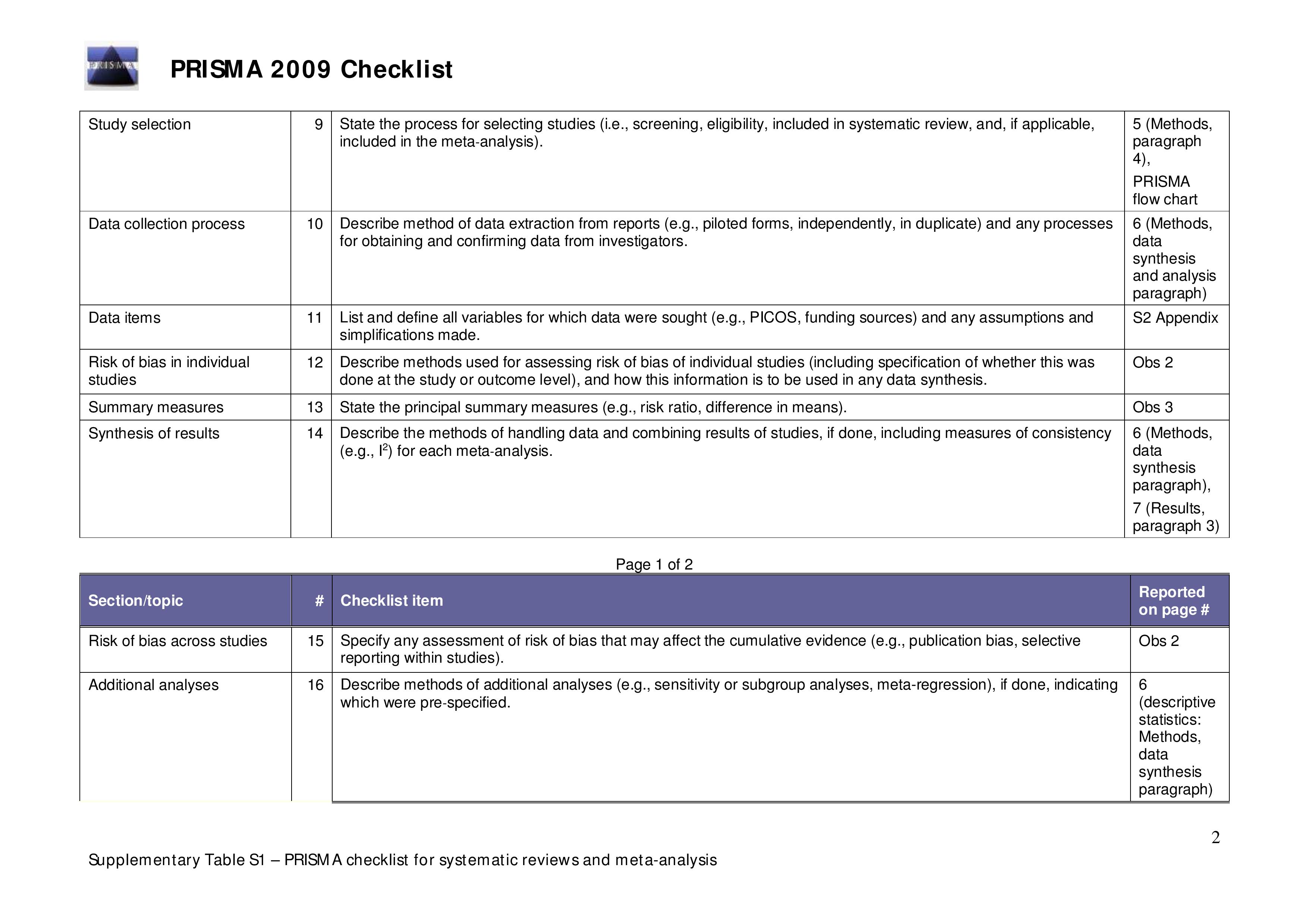

Supplement: Supplemental Material [file TEMI_A_1722035_SM1666.zip › Supplementary Material/S1_Checklist_page_002_final.jpg]

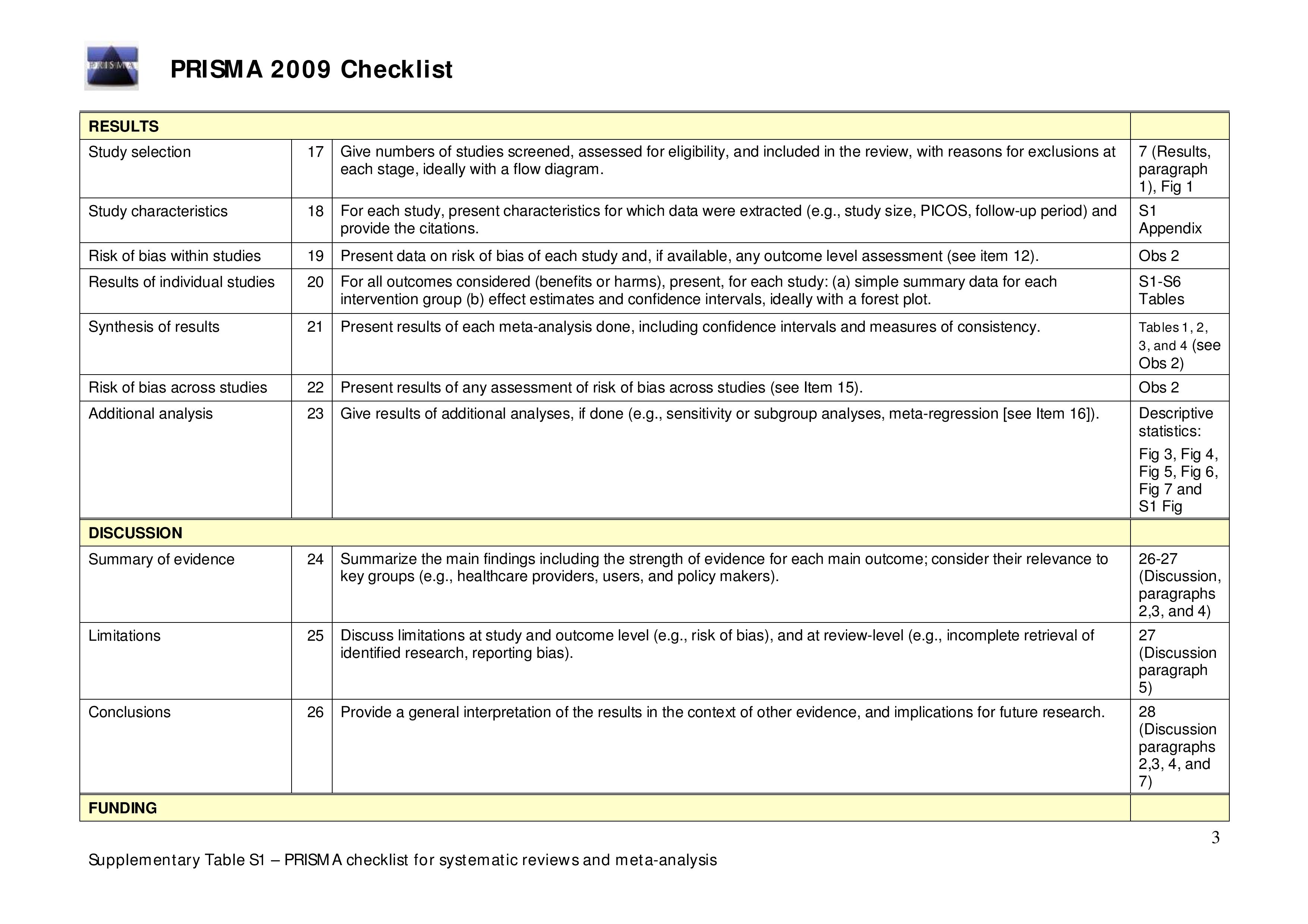

Supplement: Supplemental Material [file TEMI_A_1722035_SM1666.zip › Supplementary Material/S1_Checklist_page_003_final.jpg]

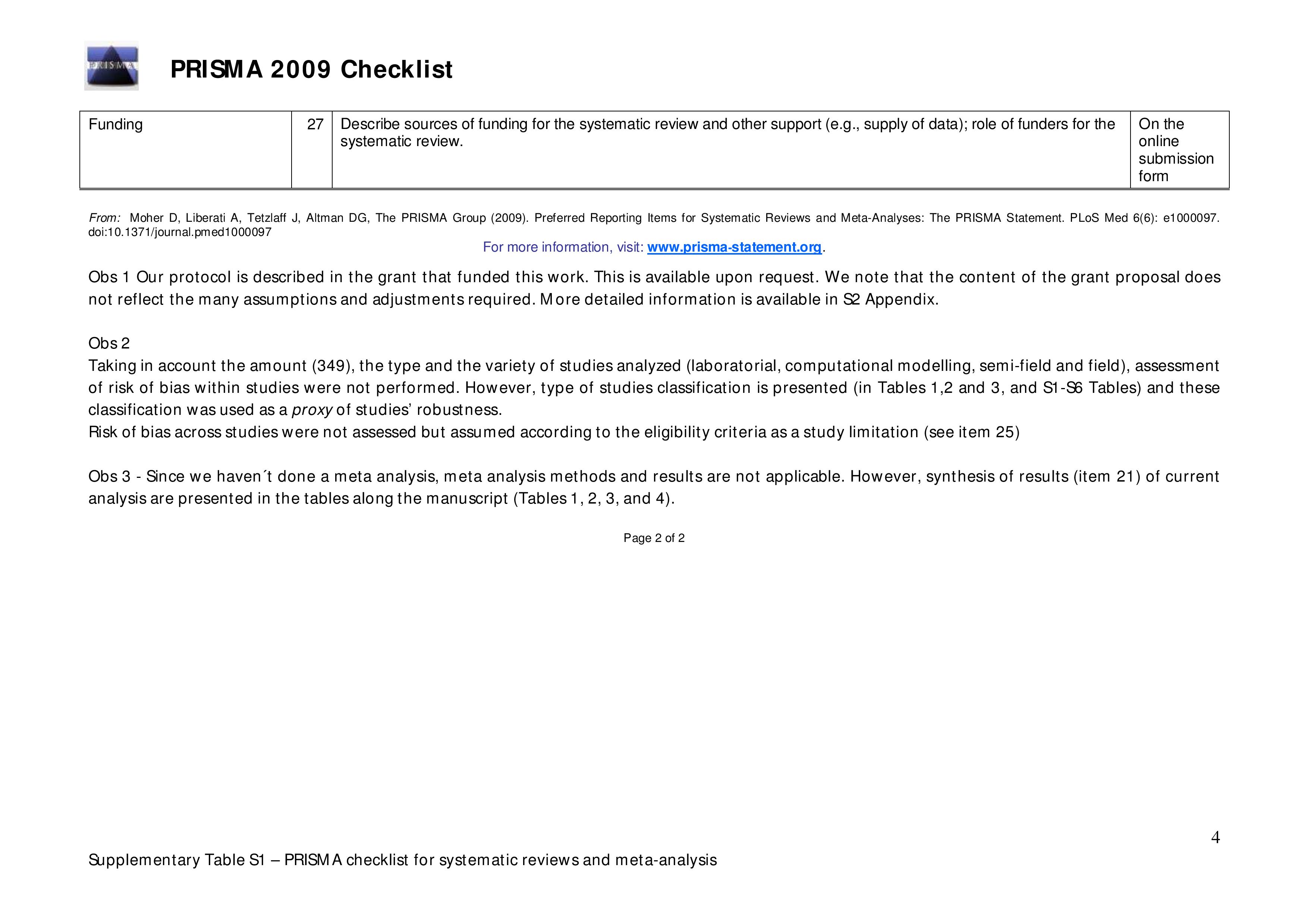

Supplement: Supplemental Material [file TEMI_A_1722035_SM1666.zip › Supplementary Material/S1_Checklist_page_004_final.jpg]

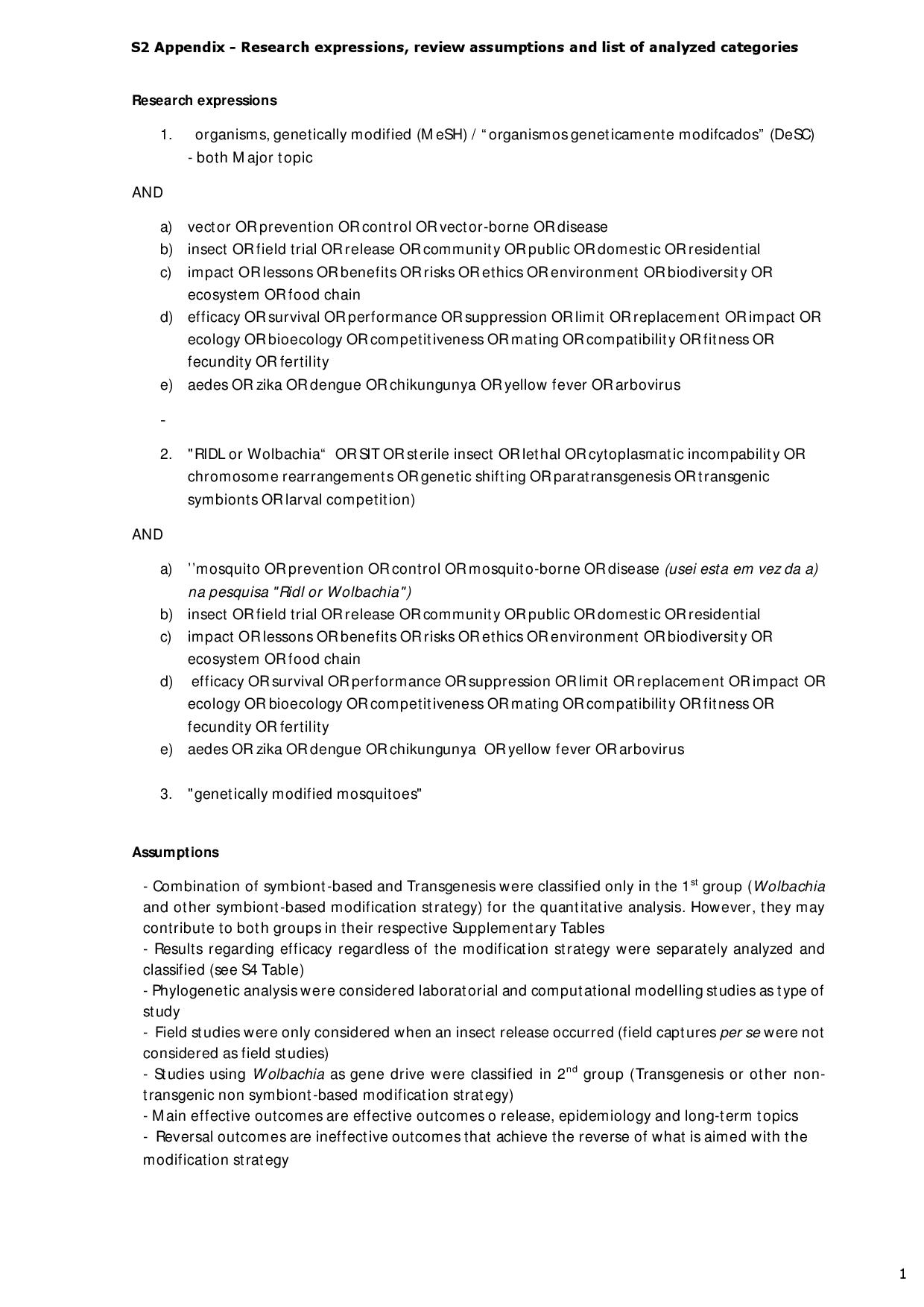

Supplement: Supplemental Material [file TEMI_A_1722035_SM1666.zip › Supplementary Material/S2_Appendix_page_001_final.jpg]

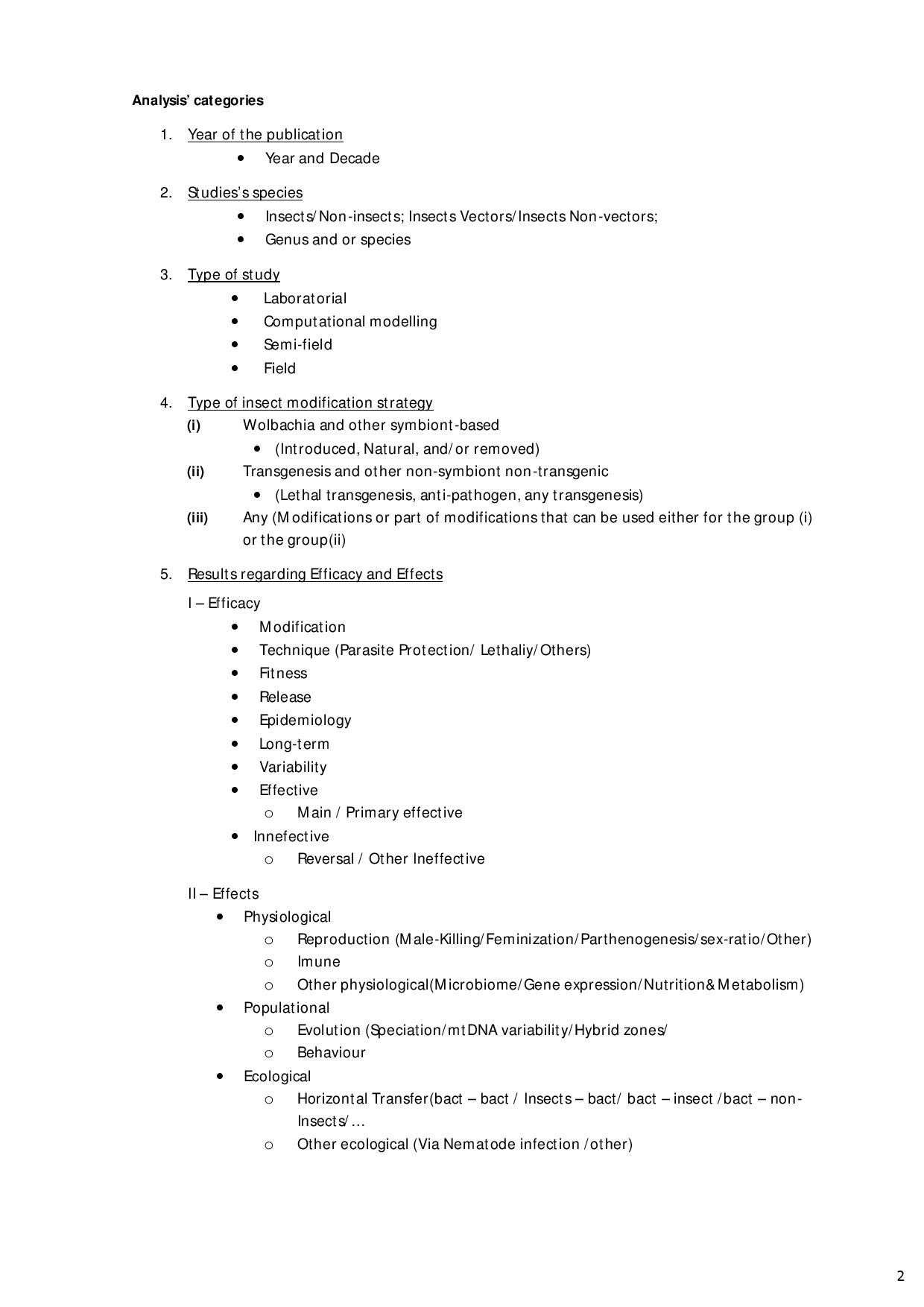

Supplement: Supplemental Material [file TEMI_A_1722035_SM1666.zip › Supplementary Material/S2_Appendix_page_002_final.jpg]
